# Transition to Piscivory Seen Through Brain Transcriptomics in a Juvenile Percid Fish: Complex Interplay of Differential Gene Transcription, Alternative Splicing, and ncRNA Activity

**DOI:** 10.1002/jez.2886

**Published:** 2024-12-04

**Authors:** Radka Symonová, Tomáš Jůza, Million Tesfaye, Marek Brabec, Daniel Bartoň, Petr Blabolil, Vladislav Draštík, Luboš Kočvara, Milan Muška, Marie Prchalová, Milan Říha, Marek Šmejkal, Allan T. Souza, Zuzana Sajdlová, Michal Tušer, Mojmír Vašek, Cene Skubic, Jakub Brabec, Jan Kubečka

**Affiliations:** ^1^ Institute of Hydrobiology Biology Centre of the Czech Academy of Sciences České Budějovice Czech Republic; ^2^ Faculty of Science University of South Bohemia České Budějovice Czech Republic; ^3^ South Bohemian Research Centre for Aquaculture and Biodiversity of Hydrocenoses, Faculty of Fisheries and Protection of Waters University of South Bohemia in České Budějovice Vodňany Czech Republic; ^4^ Institute of Computer Science Czech Academy of Sciences Prague Czech Republic; ^5^ Institute for Atmospheric and Earth System Research INAR Forest Sciences, Faculty of Agriculture and Forestry, University of Helsinki Helsinki Finland; ^6^ Institute for Biochemistry and Molecular Genetics, Centre for Functional Genomics and Bio‐Chips, Faculty of Medicine University of Ljubljana Ljubljana Slovenia

**Keywords:** brain transcriptome, cannibalism, developmental plasticity, heterochrony, (*Sander (Stizostedion) lucioperca*), snoRNA

## Abstract

Pikeperch (*Sander Lucioperca*) belongs to main predatory fish species in freshwater bodies throughout Europe playing the key role by reducing planktivorous fish abundance. Two size classes of the young‐of‐the‐year (YOY) pikeperch are known in Europe and North America. Our long‐term fish survey elucidates late‐summer size distribution of YOY pikeperch in the Lipno Reservoir (Czechia) and recognizes two distinct subcohorts: smaller pelagic planktivores heavily outnumber larger demersal piscivores. To explore molecular mechanisms accompanying the switch from planktivory to piscivory, we compared brain transcriptomes of both subcohorts and identified 148 differentially transcribed genes. The pathway enrichment analyses identified the piscivorous phase to be associated with genes involved in collagen and extracellular matrix generation with numerous Gene Ontology (GO), while the planktivorous phase was associated with genes for non‐muscle‐myosins (NMM) with less GO terms. Transcripts further upregulated in planktivores from the periphery of the NMM network were *Pmchl*, *Pomcl*, and *Pyyb*, all involved also in appetite control and producing (an)orexigenic neuropeptides. Noncoding RNAs were upregulated in transcriptomes of planktivores including three transcripts of snoRNA U85. Thirty genes mostly functionally unrelated to those differentially transcribed were alternatively spliced between the subcohorts. Our results indicate planktivores as potentially driven by voracity to initiate the switch to piscivory, while piscivores undergo a dynamic brain development. We propose a spatiotemporal spreading of juvenile development over a longer period and larger spatial scales through developmental plasticity as an adaptation to exploiting all types of resources and decreasing the intraspecific competition.

## Summary

- *Symonova* et al., Transition to piscivory seen through brain transcriptomics in a juvenile percid fish: Complex interplay of differential gene transcription, alternative splicing, and ncRNA activity
- The first characterization of developing brain gene transcription in pikeperch from its natural environment.
- The earlier, planktivorous, ontogenetic phase of pikeperch is associated with genes involved in non‐muscle myosins.
- The following, piscivorous, ontogenetic phase of pikeperch is associated with genes involved in collagen metabolism and extracellular matrix.

## 1 Introduction

Pikeperch (*Sander (Stizostedion) lucioperca* L.) belongs to the main predatory fish species in eutrophic freshwater lakes in large parts of Europe (Wysujack et al. 2002) and plays a key role in inland water bodies of the temperate zone by reducing planktivorous and omnivorous fish abundance (Dörner, Wagner, and Benndorf 1999). Pikeperch further has a high economic potential for aquaculture and fisheries in Europe (Policar et al. 2019; Colchen et al. 2020). Pikeperch spawn usually at the end of April in Czech reservoirs depending particularly on spring temperature (Jůza et al. 2023; Kratochvíl et al. 2010; Matěna, Kubečka, and Peterka 1999), whereby a guarding male stays to protect the eggs and young fry in the nest (Lappalainen, Dörner, and Wysujack 2003). Despite the relatively short spawning period in a single batch and synchronism of oocyte growth, a peculiar bimodal size distribution is common during the first growing season (Buijse and Houthuijzen 1992, Frankiewicz, Dabrowski, and Zalewski 1996; van Densen, Ligtvoet, and Roozen 1996; Saulamo, Lappalainen, and Lehtonen 2005). The size differences emerge already during the transition from pikeperch embryonic to larval development, i.e. during the switch to the exogenous feeding (Franz, Lewerentz, and Grunow 2021). After the onset of the exogenous feeding, all young‐of‐the‐year (YOY) pikeperch feed on zooplankton (Densen 1985, 1996; Ginter et al. 2012). Later, another transition starts whereby some individuals become piscivorous and demersal in the littoral while others, especially in the open water, remain planktivorous (Frankiewicz, Dabrowski, and Zalewski 1996). As a result, two clearly distinguishable groups of pikeperch YOY despite their similar age can be found in the late summer (van Densen, Ligtvoet, and Roozen 1996). Existence of two differentially behaving subcohorts provides a model system of consecutive life strategies studied in North American and European freshwater perciform fishes (Aalto and Buck Newsome 1990; Uphoff et al. 2019; Kubečka and Švátora 1993; Čech et al. 2005; Tesfaye et al. 2024). However, several crucial questions are linked to mechanisms that govern the exact timing of the transition from zooplanktivory towards piscivory and cannibalism. First, zooplankton availability reduces considerably with the onset of winter (Lampert, Lampert, and Larssona 2010) and switching to piscivory is a matter of surviving harsh winter conditions (Huss et al. 2008). Second, the earlier transition to piscivory accelerates the overall growth rate and decreases the mortality from predators (Buijse and Houthuijzen 1992, Lappalainen et al. 2000). Hence, the early transition to piscivory increases the individual fitness immensely. Till early autumn, zooplanktivorous fingerlings still can catch up with their faster siblings although their chances to survive can be a matter of further debate. The intra‐cohort size differences stimulate cannibalism already in pikeperch larvae and during the later development (Colchen et al. 2019, 2020; Lund and Steenfeldt 2011; Szczepkowski et al. 2011). Although cannibalism occurs in natural population as well, it is more pronounced in aquaculture (Yang et al. 2015; Liu et al. 2017). This is among others a reason, why the relevant studies are strongly biased towards aquaculture originating pikeperch individuals, with Franz, Lewerentz, and Grunow 2021 being one of the exceptions focusing on near‐natural conditions. This underlines the importance of pikeperch for aquaculture although understudying ontogeny of natural pikeperch populations is equally crucial.

Especially the piscivorous YOY pikeperch are likely to succeed in recruitment to the adult reproducing stock. Pikeperch recruitment at Lipno Reservoir (Czechia) gained high attention after the collapse of catches by recreational anglers in the first decade of the new millennium (Jůza et al. 2023). Hence it is desirable to investigate whether and how YOY pikeperch differentiate into the two subcohorts and what are the abundance, proportions and spatial distribution of different YOYs. While eco‐morphological traits related to the pikeperch transition to piscivory have been explored for decades (e.g. Mittelbach and Persson 1998; Sánchez‐Hernández 2020), molecular traits remain unknown. Although first studies addressing gene transcription in YOY pikeperch of different age and size have been performed, they used mRNA from pooled entire individuals and tested only panels of selected genes by qPCR (Franz et al. 2022; Tönißen et al. 2024).

Variations in gene transcription represent well‐established mechanisms shaping evolutionary outcomes of phenotypic changes (ecological divergence) already explored in fishes (e.g. Jeukens et al. 2008; Jacobs and Elmer 2021; Jacobs et al. 2020; Salisbury and Ruzzante 2022). Differences in gene transcription were recognized as major players (Schneider, Adams, and Elmer 2019; Carruthers et al. 2022), with the importance of alternative splicing recognized (Jacobs and Elmer 2021; Salisbury, Delgado, and Dalziel 2021; Singh et al. 2017). The role of variation in gene transcription during individual ontogeny has been extensively investigated in higher vertebrates, where links to diseases have been identified (Baralle and Giudice 2017; Mazin et al. 2021). In fish, several studies comparing gene transcription under different environmental conditions have been performed (e.g. Wellband and Heath 2017). Mechanisms causing variation in gene transcription involved in ontogeny relevant to this study are differential gene transcription and alternative splicing (AS; Healy and Schulte 2019; Lafuente and Beldade 2019; Steward et al. 2022; Wright et al. 2022). Differential gene transcription are quantitative changes resulting from increases or decreases in amounts of specific gene transcripts (Healy and Schulte 2019). AS causes qualitative changes by producing diverse transcripts and protein isoforms from a single gene by skipping and/or retaining some exons multiplying thus protein outcomes from a single gene (Healy and Schulte 2019). AS may contribute to the complexity of developmental programmes (Bush et al. 2017). On the other hand, certain alternatively spliced isoforms do not result in functional proteins and may downregulate gene expression by diverting a proportion of pre‐mRNA into the nonsense‐mediated decay (NMD) pathway, a mechanism known as RUST (regulated unproductive splicing and translation; Bush et al. 2017). AS plays a critical role in neurodevelopmental processes that was however studied mostly in mammals and other models (Traunmüller et al. 2023).

To understand molecular factors accompanying the switch from zooplanktivory towards piscivory, we analysed gene transcription in brain of these two highly differing YOY pikeperch subcohorts from their natural population. Our exploration revealed an interplay of differential gene transcription, alternative splicing and noncoding RNAs (ncRNA) involved in the observed eco‐morphological divergence. We provide indications of upregulated (an)orexigenic neuropeptides potentially stimulating voracity in planktivores driving their switch to piscivory. We identified a predominant activity in brain collagen and extracellular matrix suggesting an intense neuronal development and networking in piscivores.

## 2 Materials and Methods

### 2.1 Direct Ecological Surveys of Pikeperch YOY Subcohorts

**Figure 1:**
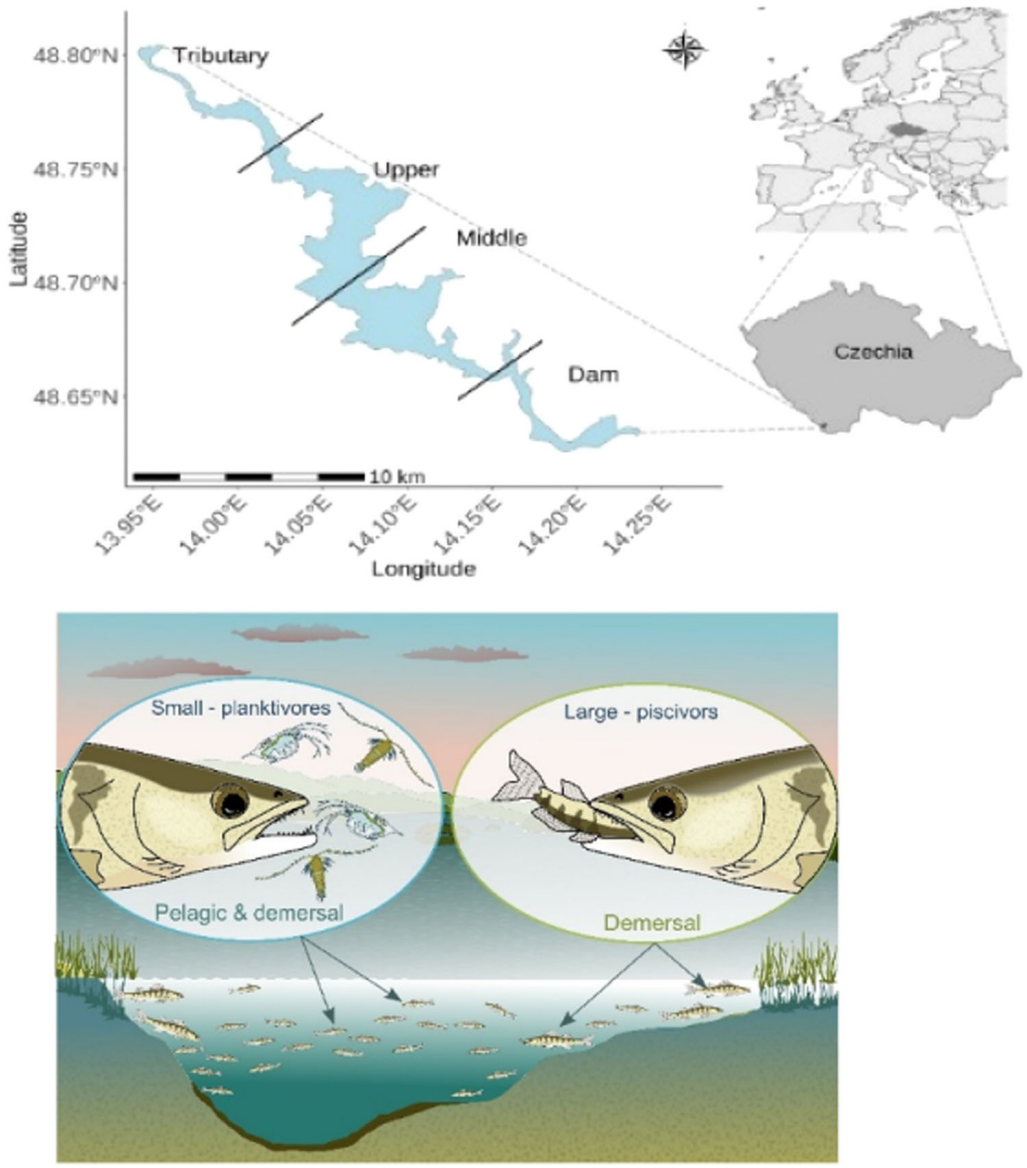
The study area and the model system. Upper panel shows the location of the Lipno Reservoir in Czechia; multimesh gillnets for the ecological survey were used in all four sampling areas, while fry trawling was done in the upper, middle and dam region (tributary part was too shallow for the trawl). Lower panel gives the scheme of spatial distribution and behaviour of the focal pikeperch subcohorts. It visualizes the model system of juvenile pikeperch distinguishing between planktivores in pelagic habitats and demersal piscivores.

The study was carried out in the Lipno Reservoir, a shallow, eutrophic water body in South Bohemia (Czechia; 720 m a. s. l.). The reservoir was constructed in 1950s for hydropower generation, flood protection, flow augmentation and recreational activities. It has a water volume of 306 million m^3^, a surface area of 46.5 km^2^, the maximum depth of 22 m, an average depth of 6.6 m (Krolová, Čížková, and Hejzlar 2012). The multimesh gillnets encompassing 12 mesh sizes according to the European standard EN 14757 and four additional larger mesh sizes (70, 90, 110 and 135 mm, Šmejkal et al. 2015) were used for sampling. The standard length (SL) of pikeperch was measured to the nearest millimetre (Table 1). To distinguish the piscivorous YOY from planktivores, we conducted stomach content analysis (unpublished data). Using the results, we verified 80 mm as the threshold SL between planktivorous and piscivorous YOY. Gillnet catches were standardized as the Catch Per Unit of Effort (CPUE) and expressed as individuals per 1000 m^2^ of the gillnet area (details on gillnet sampling in Šmejkal et al. 2015; Vašek et al. 2016). For the semiquantitative ecological survey, the reservoir was sampled by roughly 100 gillnets every year at all available benthic and pelagic habitats (depth resolution 3 m) at four sampling regions spanning the gradient between the tributary and the dam (Figure 1) in the last week of August of 2008–2010, 2012, and 2016–2023. Detailed descriptions of sampling protocol are in Kubečka et al. 2022. Age of pikeperch was determined by otolith reading *sensu* Tesfaye et al. 2023.

**Table 1:** Overview of individuals sampled for RNA‐seq, and summary of results obtained.

Subcohort	Habitat	Individuals analysed	Standard body length range	Differentially transcribed	Alternatively spliced
Planktivorous	Pelagic	3	60–68 mm	76	30
Piscivorous	Littoral/bottom	3	110–130 mm	71	

### 2.2 Samples for RNA‐Seq and RNA Extraction

For the RNA‐seq, five individuals of the planktivorous subcohort of pikeperch (*S. lucioperca* L.) were caught by night *trawling* at the Lipno Reservoir in August 2022 as described by Jůza et al. 2023. Four individuals of the piscivorous demersal subcohort of pikeperch were caught by night littoral *seining* at the Lipno Reservoir in August 2022 as described by Jůza et al. 2014. The individuals analysed in this study were too young and their sex was indistinguishable even upon dissection since their gonads were not differentiated yet. Their brains were immediately preserved with the RNA‐Later solution (Thermo Fisher Scientific, USA). A one‐side half of brain of piscivorous and the whole brain of planktivorous individuals (that were smaller) was used for each of the RNA extraction reaction to ascertain the appropriate tissue‐to‐reagent ratio. The extracted RNA was then pooled to obtain a sufficient amount of brain RNA per single individual and no tissue was left. RNA was extracted using the RNA‐Blue solution based on guanidinium thiocyanate‐phenol‐chloroform (Top‐Bio, Czechia) according to the manufacturer's instructions. This solution requires the 1:10 ratio between the tissue and the reagent. Obtained RNA was resuspended in RNAse‐free ultrapure water (Top‐Bio, Czechia), quantified with Qubit^TM^ 4 (Thermo Fisher Scientific, USA) and Qubit™ RNA Broad Range Assay Kits (Invitrogen), and stored at −80°C. RNA samples of three individuals of each subcohorts (i.e. *n* = 3 per subcohort, Table 1) with the best RNA yield and quality (RQN 8.6–10, details on sequencing statistics in Table 2) were sequenced by AZENTA (Germany) using the poly(A) enrichment and the Illumina NovaSeq pair‐end approach with the read‐length 150 bp.

**Table 2:** Sample sequencing statistics.

Sample ID	# Reads	Yield (Mbases)	Mean phred quality score	% Bases ≥ 30	RQN
Piscivor1Brain	23,198,571	6960	35.20	90.01	10
Piscivor2Brain	29,113,716	8734	35.20	90.10	9.7
Piscivor3Brain	31,641,532	9492	35.47	91.48	10
Planktivor2Brain	41,131,027	12,339	35.47	91.44	9.3
Planktivor3Brain	51,033,248	15,310	35.41	91.14	10
Planktivor4Brain	48,961,821	14,689	35.47	91.43	8.6

### 2.3 RNA‐Seq Data Filtering and Reads Mapping

Sequence reads were trimmed to remove adaptor sequences and nucleotides with poor quality using Trimmomatic v.0.36 (Bolger, Lohse, and Usadel 2014). The trimmed reads were mapped to the pikeperch reference genome SLUC_FBN_1.2 (GCA_008315115.2; Nguinkal et al. 2019) available on ENSEMBL (Cunningham et al. 2022) using the STAR aligner v.2.5.2b (Dobin et al. 2013). BAM files were generated. Sample sequencing statistics and statistics of mapping the reads to the reference genome of pikeperch are in Table 2 an Supporting Information S1: Table S1.

### 2.4 Gene Expression Analysis

Unique gene hit counts were calculated using featureCounts from the Subread package v.1.5.2 (Liao, Smyth, and Shi 2019). The hit counts were summarized and reported using the gene_id feature in the annotation file. Only unique reads that fell within exon regions were counted. The gene hit counts table was used for downstream differential expression analysis. Using DESeq. 2, a comparison of gene expression between the two subcohorts was performed with the negative binomial model. The Wald test was used to generate p‐values and Log2FoldChanges. Genes with an adjusted *p*‐value < 0.05 and absolute Log2FoldChange > 1 were called as differentially expressed genes for each comparison (Love, Huber, and Anders 2014).

### 2.5 Alternative Splicing Analysis

Differential exon usage was analysed using DEXseq (Anders, Reyes, and Huber 2012) between the two subcohorts. To estimate expression levels of alternatively spliced transcripts, the splice variant hit counts were extracted from the RNA‐seq reads mapped to the genome. Differentially spliced genes were identified for groups with more than one sample by testing for significant differences in read counts on exons (and junctions) of the genes.

### 2.6 Functional Gene Ontology, Pathway Enrichment and Network Centrality Analyses

Pathway enrichment analyses and interaction statistics were performed online using the STRING database v.12 with the zebrafish (*Danio rerio*) gene annotation (Szklarczyk et al. 2023) and the tool Cytoscape (Shannon et al. 2003). Following three sets of genes without noncoding RNAs (ncRNAs) were uploaded in the database and analysed: (i) Genes upregulated in planktivores; (ii) Genes upregulated in the piscivores; and iii. Differentially spliced genes. For these datasets, Gene Ontology (GO) was explored, local network clusters, KEGG (Kanehisa et al. 2017; Tatusov et al. 2000; Thomas et al. 2019) and Reactome (Gillespie et al. 2022) pathways were identified. Enrichment map plugin of Cytoscape was used to produce networks of the enriched pathways (Merico et al. 2010). Network centrality analysis was performed by Cytoscape and the produced network centrality metrics (degree and betweenness) were used to identify potential hub genes *sensu* Lehner et al. 2006 and Rosati et al. 2024.

### 2.7 Characterization of Noncoding RNA Transcripts and Uncharacterized Proteins

Potential RNA‐RNA interactions between ncRNAs and their host gene were *in silico* predicted with IntaRNA 2.0 (Mann, Wright, and Backofen 2017) using default setting with the following exceptions: temperature was set to 20°C that better corresponds to pikeperch body temperature in summer when the samples were collected (instead of 37°C used for mammals) and minimal number of base pairs in seed was set to 8 (instead of 7). Five best hits for each ncRNA sequence were evaluated. Predictions of the secondary structure with minimum free energy (MFE) for ncRNA transcripts were performed with the online tool RNAfold WebServer 2.5.1. using parameters RNAfold ‐p ‐d2 –noLP (Lorenz et al. 2011). Long ncRNA transcripts were characterized in the ncRNA sequence database RNAcentral. org version v22 (Sweeney et al. 2019). RNA central is a free, public resource offering access to a comprehensive up‐to‐date set of ncRNA sequences provided by a group of 51 expert databases representing a broad range of organisms and RNA types. Unknown proteins were characterized by sequence similarity‐based search with AlphaFold2 (Akdel et al. 2022) and BLAST(n) (Johnson et al. 2008).

## 3 Results

### 3.1 Ecology of the Analysed Subcohorts

**Figure 2:**
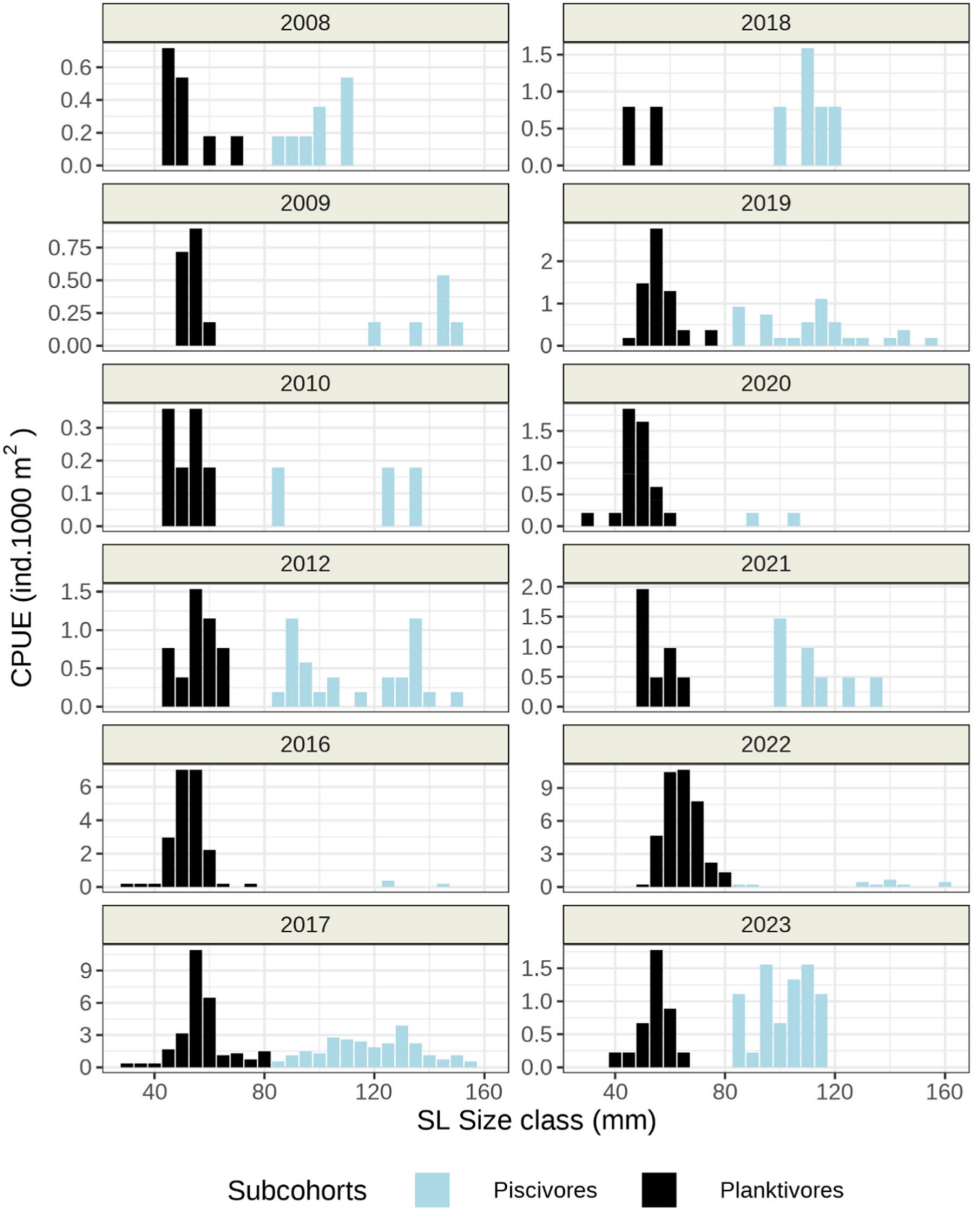
Distribution of size classes of YOY pikeperch expressed as Catch Per Unit of Effort (CPUE, individuals per 1000 m^2^of gillnets exposed per night) as collected in the Lipno Reservoir in August of the given year (years 2011 and 2013–2015 are missing). The two subcohorts analysed in this study are planktivores in black and piscivores in cyan. Note different scales of Y‐axis reflecting different abundance of YOY pikeperch in individual years.

**Figure 3:**
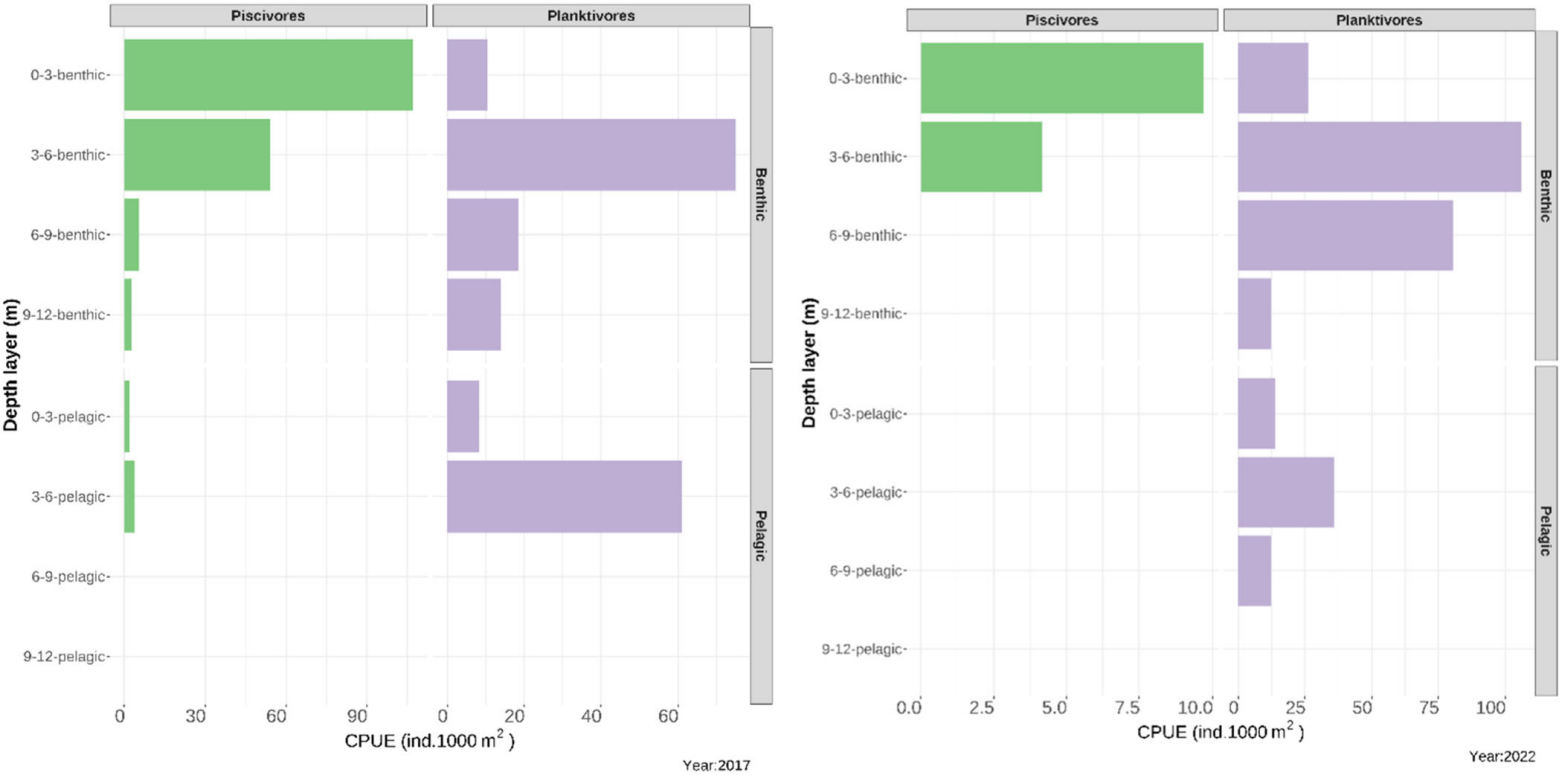
Spatial distribution of the two YOY pikeperch subcohorts in the Lipno Reservoir expressed as Catch Per Unit of Effort (CPUE) as collected in August of year 2017 (left) and 2022 (right). Piscivores are in green, planktivores in violet. The right‐hand panels discern the benthic (upper) and pelagic (lower) habitats. The left‐hand axes discern depth for each of the habitat.

Gillnet samples of YOY pikeperch differed in abundance expressed as the Catch Per Unit of Effort (CPUE) and size distributions among years (Figure 2). Two distinct size classes corresponding to the planktivorous and piscivorous subcohort were revealed in most of years. Abundant year classes of pikeperch hatched especially in 2016, 2017 and 2022. In all years except 2018, an abundant subcohort of planktivorous pikeperch with length frequency resembling the normal standard length (SL; Figure 2) distribution was present. YOY individuals larger than planktivores ( > 80 mm SL) were usually less abundant and their sizes ranged relatively randomly between 90 and 200 mm. In some years, several length frequency peaks of piscivorous YOY pikeperch were recorded. Habitats occupied by the two subcohorts overlapped only slightly (Figure 3). Piscivorous individuals were mostly missing in pelagic habitats and dominated in the littoral benthic habitat with the depth range 0–3 m. Planktivorous YOY inhabited both benthic and pelagic habitats and their peak abundance occurred in the depth range of 3–6 m. Since the benthic CPUE was slightly higher and pelagic habitats have much larger volumes than the benthic ones, the total abundance of pelagic YOY in the reservoir was higher. The distribution pattern according to the gillnet catch is shown in Figures 2 and 3.

### 3.2 Differential Gene Transcription Between the Two Subcohorts and Juvenile Brain Transcription

**Figure 4:**
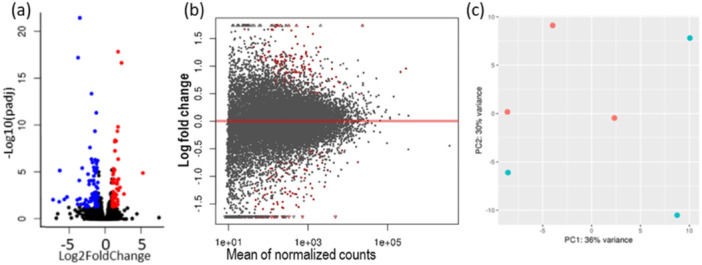
Overview of transcriptomic results between the two subcohorts. (a) The Volcano plot visualizes the global transcriptional change across the subcohorts. All genes are plotted, each data point represents a gene. The Log2FoldChange of a gene is on the x‐axis and the −Log10 of its adjusted *p*‐value (*p*adj) is on y‐axis. Genes with the *p*adj value < 0.05 and a Log2FoldChange > 1 were upregulated in piscivores (red dots). Genes with the padj < 0.05 and a Log2FfoldChange < −1 were upregulated in planktivores (blue dots). (b) The MA plot visualizes the LogFoldChange in the mean expression between the subcohorts (M) compared with mean of normalized count of each gene (A). It is focused on the dispersion of points around the red central line. It provides insights into the variability of gene transcription—points scattered around the central line have no significant differential transcription. (c) The PCA depicts the transcriptomic similarity within and between the subcohorts, red dots are planktivores, cyan dots are piscivores.

RNA‐seq yielded 20,721 transcripts in three individuals for each subcohort. Of them, 148 transcripts were significantly differentially transcribed (DT): 76 transcripts were significantly upregulated in planktivores, while 72 transcripts were significantly upregulated in piscivores (Figure 4, lists of transcripts Supporting Information S1: Table S2). In planktivores, eight noncoding RNA (ncRNA) were among the significantly upregulated transcripts. Five of them were so far uncharacterized ncRNAs and three were identified as snoRNA U85 (details below). A single uncharacterized ncRNA was identified among the transcripts significantly upregulated in piscivores. Furthermore, two uncharacterized protein‐coding genes were upregulated in planktivores, LOC116046488 and LOC116047371. The former transcript was assigned to myosin based on its conserved domains and protein similarities. The latter transcript has a potential to code for a 445‐amino acid long protein containing a DNA‐binding (BEN) domain. However, the BLASTN search revealed its sequence similarity with a teleost‐specific ncRNA, hence its function remains unclear. No uncharacterized protein‐coding gene transcript was upregulated in piscivores. Finally, following transcripts coding for proteins with functions unclear or unknown in fish (developing) brain were upregulated in piscivores: for example *Tektin 3*, *Transgelin*, *Lumican*, *Protocadherin 12*, while two transcription factors (TF) were upregulated in planktivores: fosab and junba.

Transcriptomes of planktivores were dominated by three different transcripts of the heavy and light chain of non‐muscle myosins (NMM; *Myha, Myl*), three troponins (*Tnnc2*, *Tnni2a*, *Tnnc3a*) and two creatine kinases (*Ckma*, *Ckmab*), all functionally related with the NMMs. Myosin genes had the highest exon number in planktivores. Further significantly upregulated transcripts not directly related to NMMs were pro‐melanin‐concentrating hormone like (*Pmchl*, Log2FoldChange −7,25), G protein‐coupled receptor 151 (*Gpr151*, Log2FoldChange −3.51), pro‐opiomelanocortin like (*Pomcl*, LOC116044757, Log2FoldChange −2.94), and Peptide YYb (PYYb, Log2FoldChange −2.85).

In piscivores, the significantly upregulated transcripts were genes for collagens: Collagen I α1a, α 1b and α2, Collagen V α2, Collagen VI α1, α2, α3, and Collagen XVI α1. Collagen genes had the highest exon number in piscivores. Numerous transcripts were functionally associated with collagens as shown by the gene set enrichment analysis below. The most upregulated were transcripts of two coagulation factors, F13A1 and F7I (Log2FoldChange 2.63 and 5.26, respectively).

**Figure 5:**
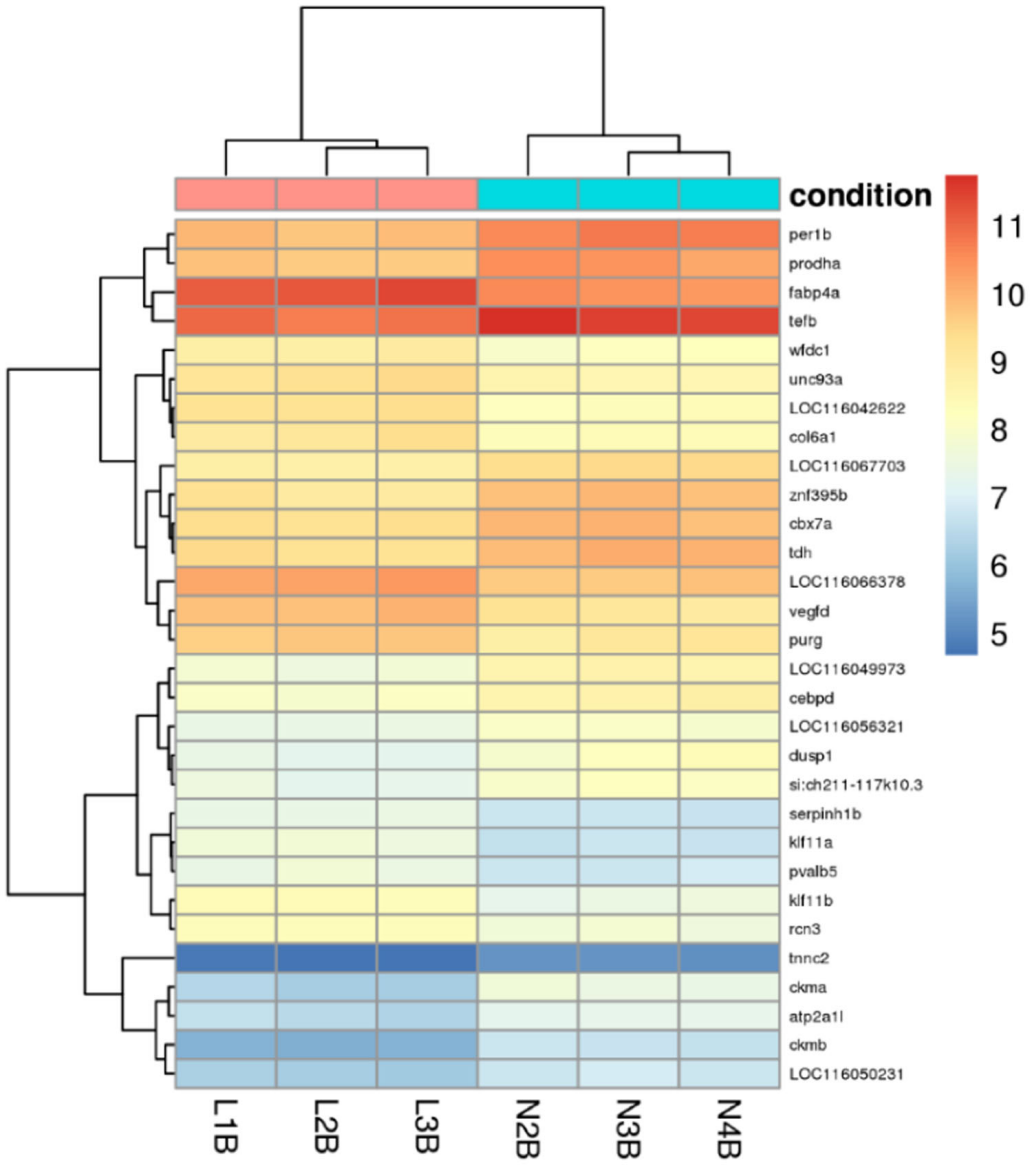
Bi‐clustering heat map of differentially transcribed genes. This analysis visualizes the transcription profile of the top 30 genes sorted by their adjusted p‐values and clustering both the samples and the genes (three “L” labelled samples refer to Littoral piscivores, three “N” samples refer to Normal planktivorous fingerlings). Yellow to red colours indicate higher relative transcription, blue colours indicate lower relative transcription. Coregulated genes across the subcohorts are shown by the tree on the left‐hand side.

Despite the significantly upregulated transcripts in both subcohorts, the overall transcription shows a certain level of variability among the three replicas of each subcohort and similarity between the subcohorts (Figure 4). The top 30 most differential gene transcripts are visualized on Figure 5.

The highest transcription based on the gene hit counts was recorded in ependymin, *Epd*, (in planktivores 249053.20, 170892.62, 171615.17, and in piscivores 322092.02, 337401.67, 486355.69). Further transcripts related to feeding behaviour and predation (but not significantly differentially transcribed) selected from the entire set of 20,721 transcripts based on literature review are in Appendix 1. This list provides a reference of genes transcribed in the developing pikeperch brain and an overview of transcription values for the differentially expressed genes.

### 3.3 Pathway and Gene Set Enrichment Analyses and Highly Connected Genes

Functional annotation of differentially transcribed genes (DTG) revealed different pathways enriched in planktivores in the comparison with piscivores (Tables 3, 4, 5, 6). Although fewer DTGs were identified in piscivores, significantly more Gene Ontology (GO)‐terms and categories are represented in this subcohort.

**Table 3:** Interaction statistics at default settings with STRING db version 12 (Szklarczyk et al. 2023).

	Input Genes	# nodes	# edges o/e	KEGG networks	Reactome networks	*p*‐value adj
Planktivorous	64	62	81/14	0	2	< 1.0e‐16
Piscivorous	71	67	59/6	2	23	< 1.0e‐16

**Table 4:** Functional enrichments of protein sets according to Gene Ontology (GO) in piscivores.

Biological processes				
GO‐term	Description	Count in network	Strength	FDR
GO:0043589	Skin morphogenesis	3 of 13	1.97	0.0140
GO:0003433	Chondrocyte development involved in endochondral bone morphogenesis	3 of 18	1.82	0.0219
GO:0030199	Collagen fibril organization	7 of 52	1.73	4.57e‐07
GO:0060351	Cartilage development involved in endochondral bone morphogenesis	4 of 32	1.7	0.0040
GO:0032963	Collagen metabolic process	4 of 57	1.45	0.0213
GO:0030198	Extracellular matrix organization	12 of 247	1.29	1.89e‐08
GO:0060348	Bone development	5 of 149	1.13	0.0361
GO:0051216	Cartilage development	5 of 159	1.1	0.0449
GO:0061448	Connective tissue development	6 of 195	1.09	0.0163
GO:0001501	Skeletal system development	10 of 406	0.99	0.00020

**Molecular function**				
**GO‐term**	**Description**	**Count in network**	**Strength**	**FDR**
GO:0005201	ECM structural constituent	7 of 117	1.38	7.94e‐05
GO:0005518	Collagen binding	4 of 76	1.32	0.0372
GO:0061134	Peptidase regulator activity	7 of 194	1.16	0.0011
GO:0030414	Peptidase inhibitor activity	5 of 164	1.09	0.0402
GO:0005198	Structural molecule activity	10 of 713	0.75	0.0118

**Cellular component**				
**GO‐term**	**Description**	**Count in network**	**Strength**	**FDR**
GO:0005584	Collagen type I trimer	2 of 2	2.6	0.0060
GO:0005583	Fibrillar collagen trimer	4 of 8	2.3	3.61e‐06
GO:0098644	Complex of collagen trimers	5 of 15	2.13	3.65e‐07
GO:0005581	Collagen trimer	10 of 102	1.59	1.54e‐10
GO:0062023	Collagen‐containing ECM	10 of 290	1.14	8.84e‐07
GO:0031012	Extracellular matrix	14 of 447	1.1	2.40e‐09
GO:0005615	Extracellular space	22 of 1324	0.82	2.99e‐10
GO:0005576	Extracellular region	36 of 2383	0.78	1.56e‐17

Abbreviation: FDR, false discovery rate.

**Table 5:** Reactome and KEGG pathways enriched in piscivores.

React. pathway	Description	Count in network	Strength	FDR
DRE‐2243919	Crosslinking of collagen fibrils	3 of 7	2.23	0.00017
DRE‐419037	NCAM1 interactions	4 of 13	2.09	1.26e‐05
DRE‐2022090	Assembly of collagen fibrils and other multimeric structures	7 of 24	2.07	8.30e‐10
DRE‐75892	Platelet Adhesion to exposed collagen	2 of 9	1.95	0.0183
DRE‐8948216	Collagen chain trimerization	6 of 29	1.92	7.47e‐08
DRE‐8874081	MET activates PTK2 signalling	4 of 21	1.88	5.99e‐05
DRE‐3000171	Non‐integrin membrane‐ECM interactions	4 of 21	1.88	5.99e‐05
DRE‐1474290	Collagen formation	9 of 55	1.82	7.99e‐11
DRE‐3000178	ECM proteoglycans	6 of 36	1.82	2.04e‐07
DRE‐430116	GP1b‐IX‐V activation signalling	2 of 12	1.82	0.0290
DRE‐1442490	Collagen degradation	6 of 37	1.81	2.07e‐07
DRE‐1650814	Collagen biosynthesis and modifying enzymes	8 of 51	1.8	9.87e‐10
DRE‐216083	Integrin cell surface interactions	7 of 54	1.72	5.20e‐08
DRE‐76009	Platelet Aggregation	3 of 30	1.6	0.0054
DRE‐186797	Signalling by PDGF	4 of 46	1.54	0.00064
DRE‐1474228	Degradation of the ECM	7 of 90	1.49	7.92e‐07
DRE‐1474244	ECM organization	11 of 207	1.33	2.54e‐09
DRE‐114608	Platelet degranulation	5 of 110	1.26	0.00083
DRE‐381426	Regulation of Insulin‐like Growth Factor (IGF) transport and uptake by Insulin‐like Growth Factor Binding Proteins (IGFBPs)	4 of 109	1.17	0.0114
DRE‐76002	Platelet activation, signalling and aggregation	7 of 207	1.13	0.00013
DRE‐9006934	Receptor Tyrosine Kinases sign.	8 of 333	0.98	0.00019
DRE‐422475	Axon guidance	5 of 220	0.96	0.0147
DRE‐1266738	Developmental Biology	6 of 302	0.9	0.0080

**KEGG Pathways enrichment in piscivores**				
***Pathway***	***Description***	***Count in network***	***Strength***	***FDR***
dre04512	ECM‐receptor interaction	7 of 99	1.45	1.44e‐06
dre04510	Focal adhesion	9 of 256	1.15	1.65e‐06

**Table 6:** Functional enrichments of protein sets (Gene Ontology, GO) in planktivores.

Molecular Function (GO)				
GO‐term	Description	Count in network strength	Strength	FDR
GO:0004111	Creatine kinase activity	2 of 7	2.07	0.0352
GO:0000978	RNA polymerase II cis‐regulatory region sequence‐specific DNA binding	12 of 1219	0.61	0.0143
GO:0003700	DNA‐binding TF activity	16 of 1889	0.54	0.0143
GO:0000976	Transcription cis‐regulatory region binding	16 of 1894	0.54	0.0143
GO:0003690	Double‐stranded DNA binding	17 of 2035	0.53	0.0143
GO:0000977	RNA polymerase II transcription regulatory region sequence‐specific DNA binding	15 of 1796	0.53	0.0143
GO:0043565	Sequence‐specific DNA binding	17 of 2107	0.52	0.0143
GO:0000981	DNA‐binding transcription factor activity, RNA polymerase II‐specific	14 of 1822	0.5	0.0205
GO:0003677	DNA binding	21 of 3157	0.44	0.0143
GO:0003676	Nucleic acid binding	25 of 4469	0.36	0.0143
GO:1901363	Heterocyclic compound binding	34 of 7285	0.28	0.0143
GO:0097159	Organic cyclic compound binding	34 of 7373	0.28	0.0143

Reactome pathways enriched in planktivores				
**Pathway**	**Description**	**Count in network**	**Strength**	**FDR**
DRE‐390522	Striated Muscle Contraction	4 of 39	1.68	0.0034
DRE‐397014	Muscle contraction	5 of 142	1.21	0.0126

In piscivores, altogether 23 GO‐terms in all three categories, i.e. Biological processes, Molecular function and Cellular component (Table 4) were functionally enriched. Most of the GO‐terms of all categories are related to collagen and extracellular matrix (ECM). Further, 20 enriched Reactome and two KEGG pathways were identified in piscivores (Table 5). Altogether the GO‐terms and Reactome pathways involve among others: collagen organization, collagen metabolic processes, assembly of collagen fibrils, crosslinking of collagen fibrils, collagen binding, collagen formation, collage degradation, collagen synthesis, collagen trimerization. Regarding ECM, following GO‐terms and Reactome pathways were enriched in piscivores: ECM constituent, ECM, extracellular space/region, membrane‐ECM interactions, ECM degradation, ECM organization, ECM proteoglycans, ECM‐receptor interaction. One of the GO‐term of the Cellular component category enriched in piscivores is functionally linking collagen and ECM, namely collagen‐containing ECM. The STRING protein‐protein interaction (PPI) network for piscivores had 67 nodes and 59 edges with average node degree 3.06 and average local clustering coefficient 0.409 (Supporting Information S1: Figure S6). The PPI enrichment *p*‐value is < 1.0e‐16 and the network has significantly more interactions than expected. Hence, genes in piscivores have more interactions among themselves than what would be expected for a random set of proteins of the same size and degree distribution drawn from the genome. Such an enrichment indicates that the proteins are at least partially biologically connected, as a group.

In planktivores, although more significantly upregulated transcripts were identified, only a single GO category, Molecular function, was functionally enriched. The GO‐terms represented were related to nucleic acids binding (Table 5). Finally, two Reactome pathways were significantly enriched, both related with muscle contraction. The STRING interaction network for planktivores has 62 nodes and 81 edges with average node degree 2.61 and average local clustering coefficient 0.346 (Supporting Information S1: Figure S7). The PPI enrichment p‐value is < 1.0e‐16 and the network has significantly more interactions than expected.

**Figure 6:**
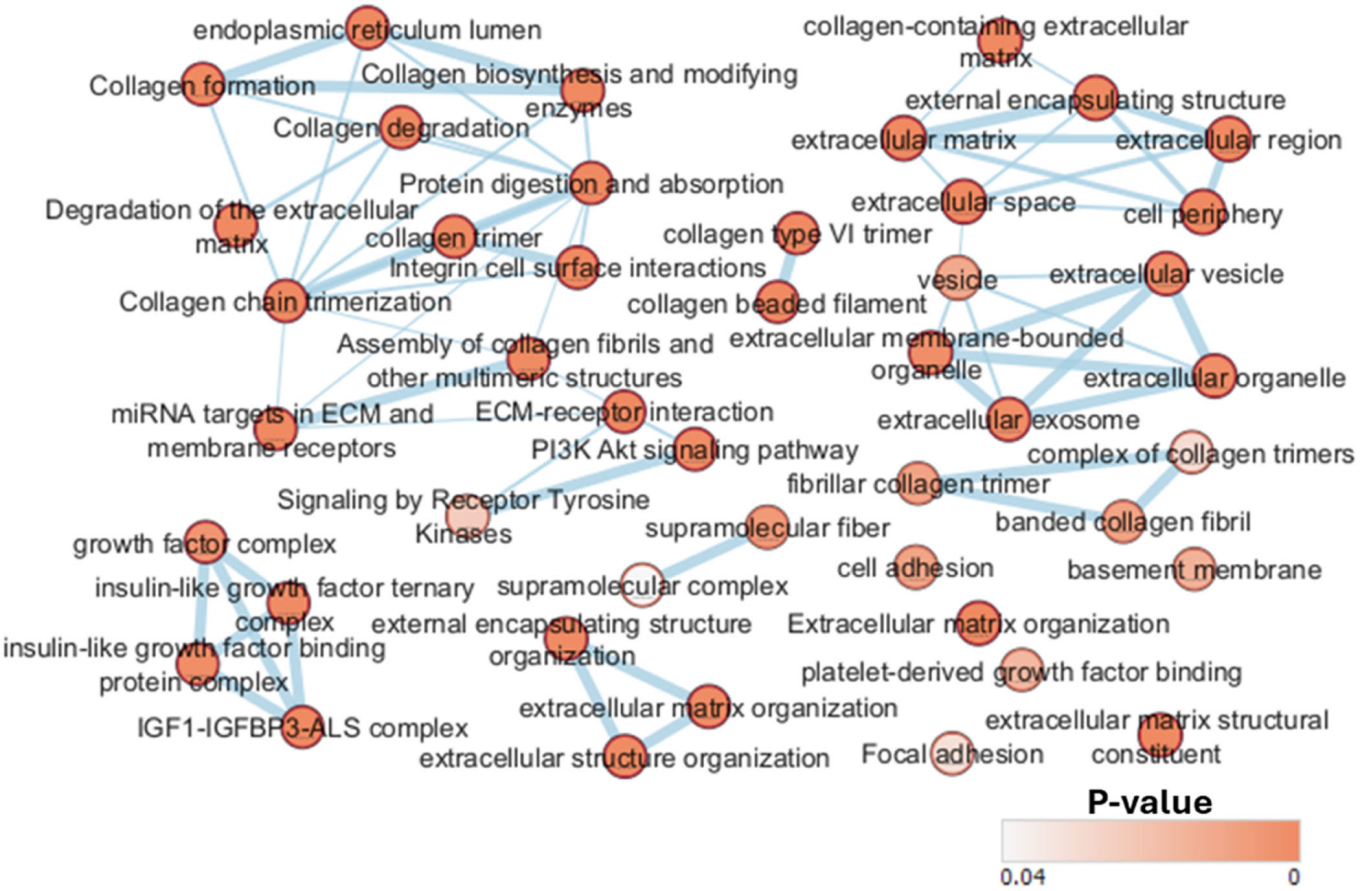
Enrichment Map of the gene‐sets enriched in piscivores forming higher order networks. No such an enrichment map was obtained for gene‐set overrepresented in planktivores. Gene‐sets are organized in a network, where each set is a node, and edges represent gene overlap between sets. In Figures 6 and 7 single genes form the nodes in both subcohorts.

The two pikeperch subcohorts significantly differ in the highly connected genes within their interaction networks based on the network centrality analysis. Myosins with their related genes (troponins, creatine kinases, parvalbumin, actin) and the TF *fosab* are highly interconnected in planktivores, collagens and their ECM related genes (serpinh1b, lumican, collagenase *mmp2*, actin, opticin, fibulin, fibronectin) are highly interconnected in piscivores (details in Supporting Information S1: File S1). The pathways and gene‐sets enriched in piscivores form higher‐order interconnected networks in the Enrichment map analysis (Figure 6). No such further interconnection was found in in planktivores, despite their transcriptomes contain more DTGs.

### 3.4 Alternative Splicing and Differential Exon Usage in the Subcohorts

**Figure 7:**
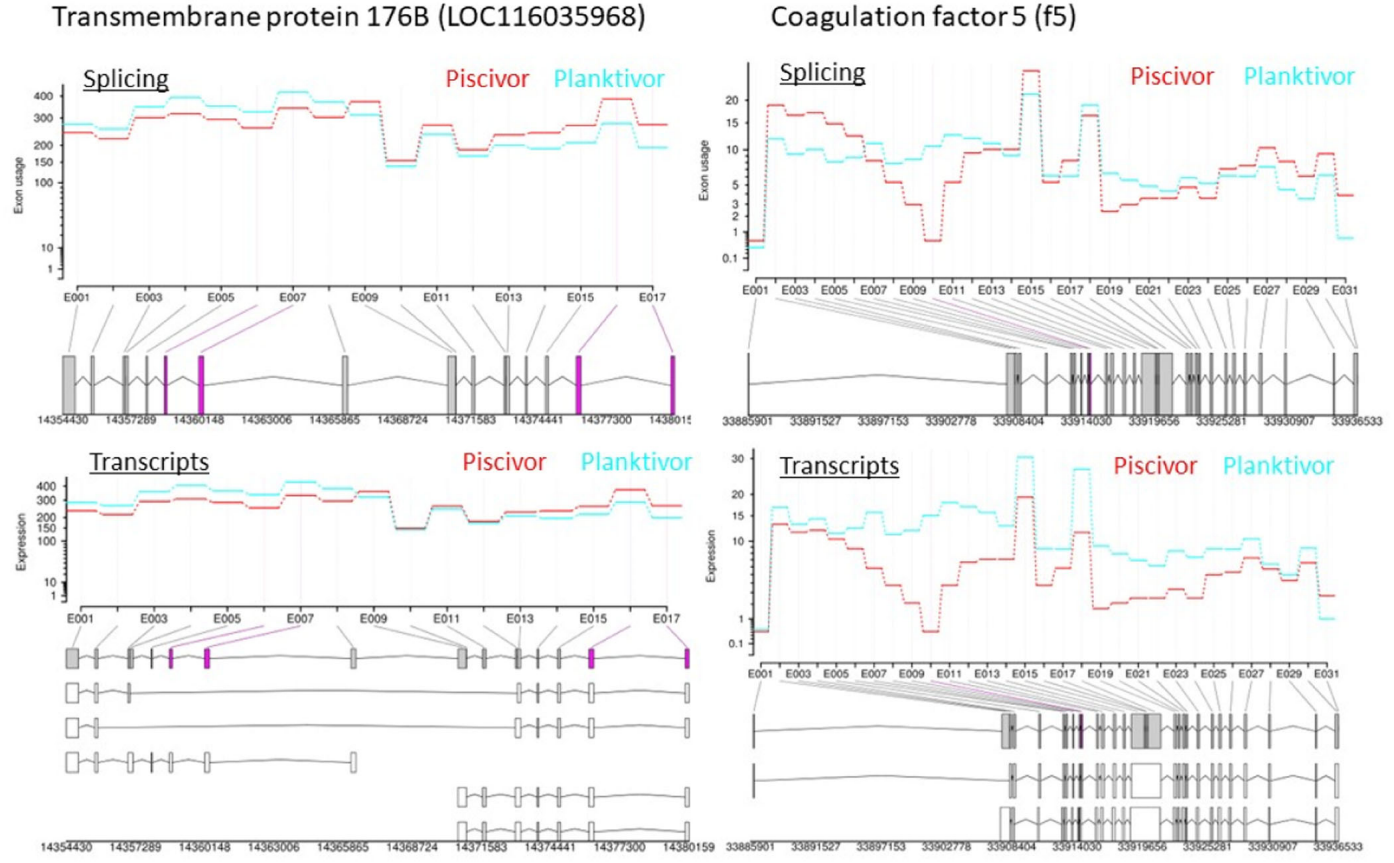
Gene models with differences and terminal and internal exons differentially spliced based on exon usage and transcripts expression. Alternative splicing patterns for Transmembrane protein 176B (LOC116035968; left panels) with four exons (6–7 and 16–17, violet bars) usage change (of total 17 exons) between the subcohorts and six transcript variants; and for Coagulation factor 5 (*f5*; right panels) with exon 10 (of total 31 exons) and three transcript variants.

The assessment of differential exon usage recorded in 32 differently spliced transcripts (DST). A single DST (*atp2a1l*, Calcium‐transporting ATPase) was at the same time significantly upregulated in the piscivores. A transcript of *acta1b*, actin alpha was DS, while acta2 was upregulated in piscivores. None of the DST was significantly upregulated in planktivores although an unconventional myosin‐IXAa belonged to the DST. The set of DSTs did not show significantly more interactions than expected (25 nodes with four edges, with the expected number of edges six). Three DSTs belong to the insulin signalling pathway that is the single significantly enriched pathway in this data set. Transcript variants with differential usage of the first and the last exons prevailed, there were also transcripts with internal exons differentially used (Figure 7). An uncharacterized ncRNA (LOC116037393 11,420 nt long with 3 exons) was among the DSTs. Finally, two so far uncharacterized DSTs were identified as putative long noncoding RNAs (lncRNA; LOC116047099 and LOC116048863), more details below. The full set of DSTs is in Supporting Information S1: Table S4.

### 3.5 Differential Transcription of Noncoding RNA Species

**Figure 8:**
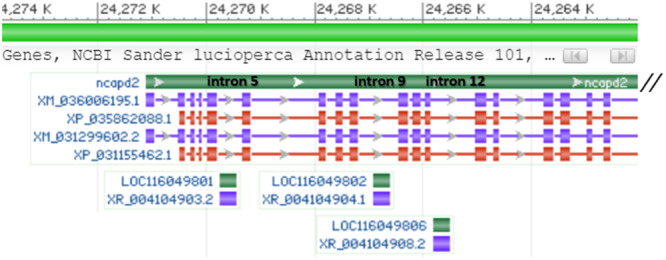
The 5′‐end adjacent part of the *Ncapd2* gene (dark green band) and its transcripts with localization of snoRNA U85 sequences (green rectangles) and their transcripts (violet rectangles) inside introns 5, 9, and 12. Visualized by NCBI Genome browser on the NC_050182.1 genomic region of the SLUC_FBN_1.2 genome assembly (GCF_008315115.2 by Nguinkal et al. 2019), annotation release 101.

Three transcripts of small nucleolar RNA (snoRNA) U85 are significantly upregulated in planktivores: (i) 315nt long XR_004104903.2 (LOC116049801), (ii) 325 nt long XR_004104904.1 (LOC116049802), and (iii) 321 nt long XR_004104908.2 (LOC116049806). These transcripts are the longest snoRNAs currently available in the NCBI for pikeperch with size range 59–219 bp (NCBI/gene). Sequences LOC116049802 and LOC116049806 show a 91.38% mutual identity. Sequences LOC116049801 and LOC116049806 show a 62.31% identity. Sequences LOC116049801 and LOC116049802 show a 61.63% identity. All snoRNAs U85 are located on the minus strand of chromosome 10 and reside in introns of a protein coding gene *Ncapd2* (Table 7, more details in the Supplementary material). The first snoRNA U85 LOC116049801 resides in intron 5 at the position 1364, the second snoRNA U85 LOC116049802 resides in intron 9 (position 4179), and the third snoRNA U85 LOC116049806 resides in intron 12 (position 5288; Figure 8). The *Ncapd2* gene codes for non‐SMC Condensin I complex subunit D2 (LOC116049696, NC_050182.1; SMC = Structural Maintenance of Chromosomes). *Ncapd2* in pikeperch is 41,060,379 nt long and can produce two transcripts of 4562 nt with 32 exons and 1399 amino acids (XM_031299602.2, XM_036006195.1). The two *Ncpad2* transcripts differ in three nt in two sites behind the position 150 nt (i.e. > 99% identity).

**Table 7:** Overview of predicted interactions between three snoRNA U85 sequences with their host gene *Ncapd2* obtained by IntaRNA 2.0 (Mann, Wright, and Backofen 2017) compared with their positions inside introns.

snoRNA U85	size	residing in *Ncapd2*	interacting with *Ncapd2*
**LOC116049801**	315 bp	intron 5	beginning of exon 6 and exon 13
**LOC116049802**	325 bp	intron 9	exon 10/exon 11
**LOC116049806**	321 bp	intron 12	exon 18 and exon 23/exon 24

The three snoRNA U85 transcripts were not transcribed equally. Transcripts LOC116049802 and LOC116049806 significantly overrepresented the transcript LOC116049801 (Table 8). The *Ncapd2* gene was, however, transcribed in both pikeperch subcohorts roughly equally. Normalized counts were 306, 241, and 202 for three piscivores and 261, 205, and 234 for three planktivores. The *Ncapd2* gene does not occur in the data set of differentially spliced genes or differentially transcribed genes.

**Table 8:** Normalized counts of snoRNA U85 transcripts in all three pikeperch individuals analysed.

snoRNA U85	Piscivor1	Piscivor2	Piscivor3	Planktivor2	Planktivor3	Planktivor4
**LOC116049806**	98.84	161.7	200.5	303.6	437.6	389.4
**LOC116049802**	121.4	142.4	347.2	420	638.7	513.3
**LOC116049801**	7.06	2.41	6.8	26.3	23.9	31.9

Beside the three snoRNA U85 transcripts, five ncRNA transcripts were significantly upregulated in planktivores, all of them so far uncharacterized (LOC116048748 long 4274 nt with 6 exons, LOC116051512 long 6586 nt with 3 exons, LOC116043730 long 27,749 nt with 9 exons, LOC118494112 long 54,543 nt with 3 exons, LOC116058717 long 2214 nt and 2 exons). A single ncRNA was upregulated in piscivores (LOC116041437 long 9337 nt with 4 exons).

Finally, we identified two putative lncRNA transcripts under the “uncharacterized protein coding genes” among the differentially spliced transcripts. The pikeperch transcript LOC116047099 is 46,954 nt long with 48 exons and overlaps in the antisense direction with a putative ncRNA LOC116047103 long 758 nt with 2 exons. The pikeperch transcript LOC116047099 shows significant similarities with lncRNA in *Cottoperca gobio* (Perciformes, Bovichtidae) URS0001972E5F_56716 with identity 83.39%, query coverage 99.79%, target coverage 96.00% and gaps representing 2.34%. The pikeperch transcript LOC116048863 (long 110,487 nt with 12 exons) shows similarities with another *C. gobio* lncRNA URS0001973C36_56716 with identity 99.14%, query coverage 47.23%, target coverage 100.00% and gaps representing 0.07%.

## 4 Discussion

This study revealed that the planktivorous phase of pikeperch YOY brain development was associated with (1) non‐muscle myosin genes and related transcripts, (2) (an)orexigenic neuropeptides with so far unclear effects to appetite control across teleost fishes, (3) transcripts related to nucleic acid processing pathways and transcription factors, and (4) transcripts of several ncRNA species. The piscivorous phase was associated with networked transcripts of genes related to collagen metabolism and extracellular matrix related pathways. The differently spliced transcripts were not related to any of the above‐mentioned pathways and constituted another level of transcription regulation between the two pikeperch subcohorts under study.

The results of the differential transcription analysis and downstream analyses obtained in this study are summarized in the Table 9. The list of further transcripts related to feeding behaviour and predation (but not significantly differentially transcribed) selected from the entire set of 20,721 transcripts based on literature (Diniz and Bittencourt 2019; Volkoff 2019; Rønnestad et al. 2017; Kubra et al. 2022) is in Appendix. This list provides the first reference of genes transcribed in the developing pikeperch brain and an overview of transcription values for the differentially expressed genes.

**Table 9:** Overview of the main differences and the most relevant differentially transcribed genes and gene ontology (GO) terms and categories between the planktivorous and piscivorous phase of YOY pikeperch brain development.

	Planktivores	Piscivores
**Upregulated GO categories**	Molecular functions	Molecular functions
		Biological processes
		Cellular components
**Upregulated GO terms**	Non‐muscle myosins‐related pathways	Collagens‐related pathways
	D/NA/RNA‐processing pathways	Extracellular matrix‐related pathways
**(An)orexigens**	Pro‐opiomelanocortin	
	Pro‐melanin‐concentrating hormone	
	Peptide Yyb	
**Hub‐genes**	Non‐muscle myosins‐related genes	Collagens‐related genes
	*Fosab*	Extracellular matrix‐related genes

### 4.1 Integration of Ecology, Evolutionary and Developmental Biology and Comparative Transcriptomics

Our results show two discrete size classes of YOY pikeperch co‐existing in only partly spatiotemporally overlapping habitats in late summer in the Lipno Reservoir during the last two decades. Such size classes probably originate earlier in pikeperch ontogeny (Franz, Lewerentz, and Grunow 2021; Tönißen et al. 2024) and diverge with age as shown here. The individual's ontogeny is a complex process depending on time, whereby evolution favours traits increasing individual's abilities to cope with its environment (Vilizzi & Kováč 2013). In juvenile pikeperch, acceleration of development is the preferred trait enhancing its chances of survival the first winter. From the viewpoint of developmental biology, the switch to piscivory represents a short period of ontogeny, during which new organism‐to‐environment interactions are achieved (Kováč 2002). The fact that some of YOY pikeperch undergo the switch faster can be ascribed to intraspecies heterochrony, a form of developmental plasticity causing a temporary phenotypic variation. It can be considered heterochrony without an evolutionary impact, similarly as accelerated maturation in aphids or mites in presence of excessive food resources (reviewed by Kováč 2002). Should there be any evolutionary impact of heterochrony, a gradual divergence into two different ecotypes would result in the slow‐developing ecotype serving as a food source for the fast‐developing ecotype and vanishing before its origin. Still, the faster switch to piscivory should lead to an overall developmental acceleration, therefore it is unclear, why discrete size classes of YOY pikeperch co‐exist during their early ontogeny. Differential gene transcription, alternative splicing, and highly regulated activity of diverse ncRNA species in pikeperch brain before and after the switch to piscivory may indeed harbour molecular features accompanying this crucial ontogenetic transition. Until now, only adult tissues or juvenile and adult tissues under stress conditions not related to this size differentiation were explored by RNA‐sequencing (Wang et al. 2019; Żarski et al. 2020; Nguinkal et al. 2021; Schäfer et al. 2021; Topal et al. 2021; Liu et al. 2022; Verleih et al. 2023).

### 4.2 Non‐Muscle Myosins in Planktivorous Pikeperch Brain Development

Non‐muscle myosins (NMMs) and related transcripts were significantly upregulated in planktivorous pikeperch and formed a functional network. All these genes also dominated the highly connected transcripts in the network centrality analysis and can be considered hub genes of planktivores (details in Supporting Information S1: Figure S2). No NMM was upregulated in piscivores. NMMs are a diverse group of myosin isoforms occurring outside muscles. Several classes of NMMs are expressed in the nervous system, where they fulfil essential roles in development and functioning of neural tissues (Porro et al. 2021). NMMs play crucial roles in cellular processes related to development and maintenance of neural tissues in the brain (Gutzman, Sahu, and Kwas 2015, Das et al. 2023). NMMs in brain are involved in cell migration and morphogenesis, which are critical during brain development. NMMs assist in guiding neuronal migration to their destinations, contributing to the proper formation of neural circuits. NMMs participate in neurite outgrowth and guidance, influencing the extension of axons and dendrites and their navigation to establish connection with target cells. Synaptic NMMs ensure proper connectivity within neural networks. By their participation in tissue remodelling and plasticity, NMMs contribute to structural changes in the developing brain. This is essential for adapting neural circuits and connectivity in response to sensory input and experience. The actin cytoskeleton carries out cell division, migration, adhesion, and intracellular transport, that require a variety of actin binding proteins, including myosins. Class II NMMs are regulated by the actin binding protein, tropomyosin (Barua et al. 2014). NMMs control radial glial basal end feet to mediate interneuron organization (D'Arcy et al. 2023).

Only skeletal myosins were recently analysed in embryonal and larval pikeperch by qPCR from pooled entire individuals (Franz et al. 2022). We thus provide first insights into NMMs in developing pikeperch brain.

### 4.3 Collagens and Extracellular Matrix in Piscivorous Pikeperch Brain Development

Four types of collagens and numerous ECM structural constituents are significantly upregulated and included in functional networks of transcripts in piscivores. All these genes also dominated the highly connected transcripts in the network centrality analysis and can be considered hub genes of piscivores (Supporting Information S1: Figure S3). No collagen type neither ECM related transcript was upregulated in planktivores. Collagens are proteins with triple helices and cardinal components of extracellular matrices (ECMs; Hubert et al. 2009). While they only rarely occur in the adult nervous system, collagens together with other ECM molecules are the key determinants of juvenile brains participating in axonal guidance, synaptogenesis, establishment of the architecture of the brain, and Schwann cell differentiation and play active roles here (Hubert et al. 2009). Neurons, glial cells (astrocytes and microglia) and the ECM gave rise to the “tetrapartite” synaptic model (Dityatev and Rusakov 2011). Synaptic signalling involves the surrounding ECM. The ECM incorporates and stores molecular traces of both neuronal and glial activities. It further modulates function of local receptors or ion channels and send diffuse molecular signals using products of its use‐dependent proteolytic cleavage. This inclusive view helps to better understand the mechanisms underlying signal integration and long‐term homoeostatic regulation in the developing brain.

The two most significantly differentially transcribed genes in piscivores are coagulation factors VII (aka Stable factor or Proconvertin, F7I) and XIII (aka Fibrin stabilizing factor, F13A1). These proteins are crucial for neuro‐immune homoeostasis and interfere with synaptic homoeostasis in other ways than through coagulation itself. Their specific functions modulate neuronal networks, acting both on resident (neurons, astrocytes, and microglia) as well as circulating immune system cells and the *extracellular matrix* (De Luca et al. 2017, 2018). However, the factor F7I was not involved in the main Collagen‐ECM functional network by GSEA and was functionally associated only with Tissue factor pathway inhibitor, tfpi2. The factor F13A1 was weakly linked with the main network through Prolyl 3‐hydroxylase 1, P3h1, although available literature does not link this factor directly with brain or neural development yet (Muszbek et al. 2011). This indicates that this field is still highly understudied lacking many details. Coagulation and the immune system interact in physiological conditions including tissue repair, host defence, and homoeostatic maintenance. These factors are essential for neuroimmune homoeostasis of the central nervous system (CNS) by involving several cells (CNS resident cells, platelets, endothelium, and leucocytes) and molecular pathways (protease activity, complement factors, platelet granule content; De Luca et al. 2018).

Collagens were also analysed in embryonal and larval pikeperch by qPCR from pooled entire individuals (Franz et al. 2022) and in early and late larval pikeperch before onset of piscivory (Tönißen et al. 2024). However, the latter study did not identify any size‐related differences in gene transcription.

### 4.4 System of (An)Orexigenic Neuropeptides in the Planktivorous Subcohort

Pro‐melanin concentrating hormone (PMCH) constitutes a preproprotein with three protein domains that is proteolytically processed to generate three neuropeptides: melanin‐concentrating hormone (MCH), glutamic acid‐isoleucine (NEI), and glycine‐glutamic acid (NGE). MCH is an orexigenic neuropeptide that stimulates hunger, controls feeding behaviour, energy homoeostasis, and stress responses in mammals and potentially in fish (Takahashi et al. 2004; Kawauchi 2006; Berman et al. 2009). The MCH system is a robust integrator of exogenous and endogenous information ensuring an appropriate response to maintain the organism's homoeostasis in mammals and is less understood in fish (Diniz and Bittencourt 2019). Pro‐opiomelanocortin (POMC) was suggested to participate in hypothalamic integration of metabolic, endocrine and circadian signals regulating appetite (Delgado, Cerdá‐Reverter, and Soengas 2017; Diniz and Bittencourt 2019; Soengas 2021; Harno et al. 2018). There are still unclarities among different fish lineages, whether MCH is purely orexigenic (Rønnestad et al. 2017) or unclear or even anorexigenic (Volkoff 2016; Diniz and Bittencourt 2019). Both POMC and MCH were linked with fasting in fish, although in diverse reactions to it (Bertucci et al. 2019; Xu et al. 2019). In fish, MCH is involved also in the regulation of pigmentation, where its name comes from (Kawauchi 2006). Further functions were recently proposed for MCH and the MCH‐producing hypothalamic neurons in mammals. Although still considered as relatively understudied neuronal population even in mammals, MCH‐producing neurons were shown to integrate homoeostatic regulation and motivated behaviour with widespread inputs and outputs throughout the entire CNS (Concetti et al. 2024). As demonstrated in rats, MCH neurons thus orchestrate both food‐motivated appetitive and intake‐promoting consummatory processes (Subramanian et al. 2023). Since also the G protein‐coupled receptor 151 (GPR151) was significantly upregulated in planktivorous pikeperch and MCH binds to G protein‐coupled transmembrane receptors to mediate its function (Kawauchi and Baker 2004) we can assume that the upregulated *Pmchl* gives rise to MCH in planktivores. POMC, upregulated in planktivores, is another neuropeptide precursor that gives rise to several bioactive peptides, including alpha‐melanocyte‐stimulating hormone (α‐MSH). POMC is known for its role in regulating energy balance, appetite, and pigmentation. The last member of the appetite controlling peptides with transcripts upregulated in planktivores is peptide YYb (Pyyb/PYYb). Based on studies in mammals, PYYb was described as a gastrointestinal hormone (Persaud and Bewick 2014). Later, a full‐length PYYb was found in neurons of the hindbrain and its link to food deprivation was revealed in mammals (Gelegen et al. 2012) and finally in fish (Chen et al. 2014; Senzui & Fukada 2023). Afterwards, several studies explored its expression in fish under food restriction condition; eight studies reviewed by Bertucci et al. 2019 resulted in different responses. Hence, POMC and MCH functionally overlap in controlling feeding behaviour, appetite, energy expenditure and energy balance, and responses to metabolic challenges. However, knocking‐out the *Pomc* revealed an antagonistic activity between MCH and α‐MSH in the control of melanosome organization and activity regulating skin pigmentation (Madelaine et al. 2020). GPR151 alone is an orphan GPR selectively expressed in rodent and human habenular neurons, specifically regulates cyclic adenosine monophosphate levels and synaptic neurotransmission (Antolin‐Fontes et al. 2020). In zebrafish, GPR151 was found to be expressed in dorsal habenula (Broms et al. 2015).

### 4.5 Transcripts with Unknown or Unclear Function in (Developing) Fish Brain

Numerous genes already well annotated in mammals or in other models are still unknown in fish or their function is unclear in (developing) fish brain (e.g. Gpr151). This is probably, why several genes identified upregulated in the pikeperch subcohorts could not be properly linked in GSEA to other brain factors. The differentially transcribed but with unknown and/or unclear function in juvenile fish brain are: *Tektin 3* – while tektins are known in cilia and flagella from green algae to sea urchin (Bastin and Schneider 2019), tektin 3 is known merely in mouse and rat spermatozoa (Takiguchi et al. 2011; Roy et al. 2009); *Melanoregulin* with a potential role for cholesterol recognition is known in mammals but not in brain (Rout et al. 2018); *Transgelin* although known for their roles in non‐muscle cell motility during adhesion, migration, proliferation and phagocytosis, their physiological functions remain to be established (Hsieh and Jin 2023); *Lumican* is one of the major keratan sulphate proteoglycans (KSPGs) in the vertebrate cornea and sclera (Yeh et al. 2010). Lumican also mediates an inter‐axonal molecular crosstalk in the corticospinal innervation in mammals but with an unknown function in fish, where no cortex exists (Itoh et al. 2023); *Protocadherin 12* (*Pcdh12*,) is not known in fish brain or any other fish tissue, however, in brain of higher vertebrates, it is responsible for neuronal differentiation and development by contribution to the establishment of distinct neural identities and formation of diverse neural cell types. Pcdh12 further contributes to the diversity and specificity of neuronal connections essential for the precise wiring of the vertebrate brain (Fazeli et al. 2022). Protocadherins generally assist neural circuit formation and define cell adhesion properties as cell adhesion molecules facilitating interactions between cells. This is important for cell positioning, migration, and the formation of neural structures particularly during early ontogeny (Wu and Jia 2021). The proteins and their corresponding genes will be crucial targets for future studies. Finally, two transcription factors (fosab and junb) were upregulated in planktivores, however, their role in the developing fish brain remains unresolved (Kuleshov et al. 2016).

### 4.6 Consequences for YOY Pikeperch Ecology

By their shift to littoral, piscivorous pikeperch benefit from shelters availability, higher‐quality food, and a higher (more preferred) temperature. At the same time, the shift from the monotonous pelagic to heterogenous littoral requires/initiates further sensorics and behavioural changes in their brains. They also need capabilities and energy to manage to defend their newly gained position. Hence, all the above‐mentioned factors further accelerate the rate of individual's development. All these factors must be reflected in their brain transcriptome because predators need different capabilities utilizing the shelters and refining their hunting strategies to catch the prey than planktivores. On the other hand, planktivores remain in the pelagic habitat with the suboptimal temperature and low‐quality food far from being stimulated to their further development. They have just two options, either to switch to piscivory or to die during the first winter. Our results indicate, they are well‐equipped to switch to piscivory encouraged by their voracity. Upon their potential successful contacts with a fish prey their brain transcription activity would change from the non‐muscle myosins and (an)orexigens with their putative receptor Gpr151 to collagen and ECM constituents. The major external trigger is probably food because food quality belongs to the most influential epigenetic modifiers extensively driving gene transcription. In the new phase, an acceleration of brain development and growth takes place together with accelerated overall body growth, all governed by the acquired piscivory. Such a gradual switch to piscivory throughout the whole first summer as observed in the Lipno Reservoir could be an adaptive strategy enabling to fully exploit both types of food resources available, i.e. both fish and zooplankton. Abrupt switch of whole year class to piscivory could lead to exhaustion of YOY fish prey during the summer and food deficit during the winter. The spatial segregation of the subcohorts could help decrease the intraspecific competition and potential cannibalism.

### 4.7 Limitations and Perspective of This Study

Our study represents the very first endeavour to reveal molecular mechanisms accompanying the transition from planktivory towards piscivory in a still non‐model fish species and in individuals originating from natural habitats. Moreover, transcriptomics of juvenile fish brain generally is still in its infancy. For these reasons, our study faces several challenges. First, the insufficient annotation of genes and noncoding elements hinders more detailed understanding molecular processes in the developing pikeperch brain in comparison with more explored species and particularly mammals. Therefore, we do not know yet the exact role of several gene products in the developing pikeperch brain. However, since pikeperch genomics and transcriptomics has been gaining in its importance, new genomics resources are expected to emerge. Second, sex determination in such young individuals is impossible because of absence of any sex dimorphism traits as well as molecular sex markers and differentiated sex chromosomes that might have been utilized. Since individuals analysed in this study were too young to produce any sex‐related products because of their gonads were not yet differentiated, we do not expect any serious interference of sex with our results. On the other hand, there might be some sex‐related differences e.g. in the timing of the onset of piscivory. Third, we were not able to evaluate the exact age of individuals in the two subcohorts. Theoretically, the smaller planktivorous fish could hatch slightly later in the year and therefore could be slightly younger than the piscivorous individuals. On the other hand, it is generally accepted that the two subcohorts come from the main spring spawning of the pikeperch and are of similar age (Buijse and Houthuijzen 1992; Frankiewicz, Dabrowski, and Zalewski 1996; Lappalainen, Dörner, and Wysujack 2003; Huss, Van Kooten, and Persson 2010).

On the other hand, our results lay the foundation for disentanglement of developmental changes occurring in cultured pikeperch, where heavy modifications to the natural conditions are anticipated and where cannibalism represents a serious problem. Finally, our results are crucial for understanding pikeperch life‐history traits facing the climate change, where temperature plays an important role subcohort development – the higher temperature and the higher temperature for a longer period may prolong the chances of planktivores to accomplish their transition to piscivory.

## 5 Conclusions

This study provides the first differential transcriptomics and alternative splicing resources for juvenile pikeperch brain related to its transition from planktivory to piscivory based individual samples (i.e. not on samples pooled from more individuals). The brain tissue is suitable to address the eco‐physiological question tightly linked with ontogeny and developmental plasticity and the obtained data revealed the complexity of brain gene regulation. We identified only little overlap between the identity and biological functions of differentially transcribed and differentially spliced genes. This suggests that these two regulatory mechanisms act on different cellular traits to complementarily alter the resulting phenotype. Finally, pikeperch proved as a useful model to study the process of the gradual transition towards piscivory throughout the whole first summer of life.

## Author Contributions

Radka Symonová and Jan Kubečka conceived the project. Jan Kubečka, Marek Brabec, Tomáš Jůza, M. Te., M. Tu., Daniel Bartoň, Petr Blabolil, Vladislav Draštík, Luboš Kočvara, Milan Muška, Marie Prchalová, Milan Říha, Marek Šmejkal, Allan T. Souza, Zuzana Sajdlová, Michal Tušer and Mojmír Vašek performed the long‐term ecological observations on the co‐occurrence of both pikeperch ecotypes and enabled the tissue field sampling. Radka Symonová performed the tissue field sampling and conducted the laboratory and bioinformatic analyses. Jan Kubečka, M. Te. and Marek Brabec performed the quantitative evaluation and interpretation of the long‐term ecological data. Cene Skubic supported the authors in transcriptomic data processing. Radka Symonová and Jan Kubečka wrote the initial manuscript. All authors contributed to the writing of the final manuscript.

## Conflicts of Interest

The authors declare no conflicts of interest.

## Supporting information

Supporting information.

Supporting information.

Supporting information.

## Data Availability

The data that supports the findings of this study are available in the supplementary material of this article. The RNA‐seq data used for this project will be available on ENA under the BioProject XXXX with the following Run accessions: will be available later. Transcripts counts for expression analysis and exon count tables for the exon usage will be deposited in the Dryad repository.
