# Supplementary material for: Transition to Piscivory Seen Through Brain Transcriptomics in a Juvenile Percid Fish: Complex Interplay of Differential Gene Transcription, Alternative Splicing, and ncRNA Activity
Source: J Exp Zool A Ecol Integr Physiol. 2024 Dec 4;343(2):257–77. doi: 10.1002/jez.2886 (PMC11788885; doi:10.1002/jez.2886)
Supplement: Supplementary file 1 — Supporting information. [file JEZ-343-257-s002.docx]

**Supplementary Material**

**Transition to piscivory seen through brain transcriptomics in a juvenile percid fish: Complex interplay of differential gene transcription, alternative splicing, and ncRNA activity**

Radka Symonová | Tomáš Jůza | Million Tesfaye |Marek Brabec | Daniel Bartoň | Petr Blabolil | Vladislav Draštík | Luboš Kočvara | Milan Muška | Marie Prchalová | Milan Říha | Marek Šmejkal | Allan T. Souza | Zuzana Sajdlová | Michal Tušer | Mojmír Vašek | Cene Skubic | Jakub Brabec | Jan Kubečka

**Table of Contents**

**Supplementary results and discussion**

**1. Exon numbers and chromosomal locations of DT and DS genes**

**2. Characteristics of highly connected genes in the pikeperch subcohorts**

**3. Details on snoRNA U85 and its target gene *Ncapd2***

**4. Pathway enrichment analysis with STRING**

**5. Variants analysis performed on transcriptomics data with CLC Genomics Workbench**

**Supplementary figures**

**Figure S1** Exon counts and chromosome locations of differentially transcribed and spliced genes

**Figure S2** Network centrality analysis in planktivores and planktivores-specific hub genes

**Figure S3** Network centrality analysis in piscivores and piscivores-specific hub genes

**Figure S4** Minimal free energy (MFE) per query-target pair in snoRNA and *Ncapd2*

**Figure S5** Predicted MFE secondary structure of three snoRNA U85 upregulated in planktivores

**Figure S6** Interaction network of transcripts upregulated in the piscivorous pikeperch subcohort

**Figure S7** Interaction network of transcripts upregulated in the planktivorous pikeperch subcohort

**Supplementary tables**

**Table S1** Statistics of mapping the reads to the reference genome of pikeperch

**Table S2** Transcripts differentially transcribed between the two subcohorts with their basic features (Excel file, sheet 1)

**Table S3** Differentially spliced transcripts (genes, ncRNAs, lncRNAs) (Excel file, sheet 2)

**Table S4** Intersection between the DT and DS transcripts (Excel file, sheet 3)

**Table S5** Normalized counts of snoRNA U85 transcripts in all pikeperch individuals analysed

**Table S6** Single nucleotide variants in individuals analysed

**Supplementary references**

**Supplementary results**

**1. Exon numbers and chromosomal locations of DT and DS genes**

The average exon number was 13 for differentially transcribed genes. In planktivores, the average exon count was 11 (with 1 - 119 exons), whereby genes encoding myosins had the highest exon counts. The average exon count in piscivores was 14 (1 - 72 exons) with genes for collagen reaching the highest exon counts. The average exon number in the differentially spliced genes was 20 (5 - 55 exons; Figure S1a). The differentially transcribed and spliced genes occupy different chromosomes as shown in Figure S1b.


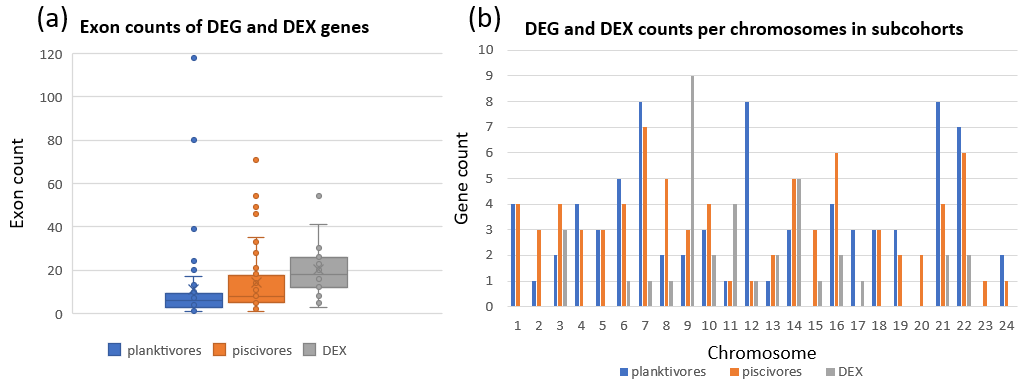
**Figure S1** Exon counts and chromosome locations of differentially transcribed and spliced genes. (a) Exon counts of differentially transcribed genes in planktivores and piscivores and in differentially spliced genes. (b) Counts of differentially transcribed genes in planktivores and piscivores and of differentially spliced (DEX) per each chromosome.

**2. Characteristics of** **highly connected genes in the pikeperch subcohorts**

The network centrality analyses revealed different highly connected sets of genes in the two investigated pikeperch subcohorts (Figure S2-S3).

# The network centrality analysis in planktivores highlighted 25 genes (Figure S2). These genes include myosins (*Mylpfa* - myosin light chain, phosphorylatable, myha – myosin heavy chain A) and their related genes for troponins (*Tnnc2*, *Tnnt3a* - troponin T type 3a, *Tnni2a2* - troponin I), actin (*Acta1b*), creatinine kinases (*Ckma, Ckmb*), parvalbumin 2 (*Pvalb2*). The highest degree centrality had Mylz3 (myosin light chain 3). Two of the (an)orexigenic factors, *Pomcl* (*Pomca* in zebrafish, whose gene annotation was used in the tool) and *Pmchl* belonged to the highly connected genes. Three transcription factors (TFs) were among the highly connected genes (*Junba* - TF jun-B, *Klf15* - Krueppel-like factor 15, and an oncogene *Fosab* (v-fos FBJ murine osteosarcoma viral oncogene homolog Ab), which had the highest value of betweenness.

**
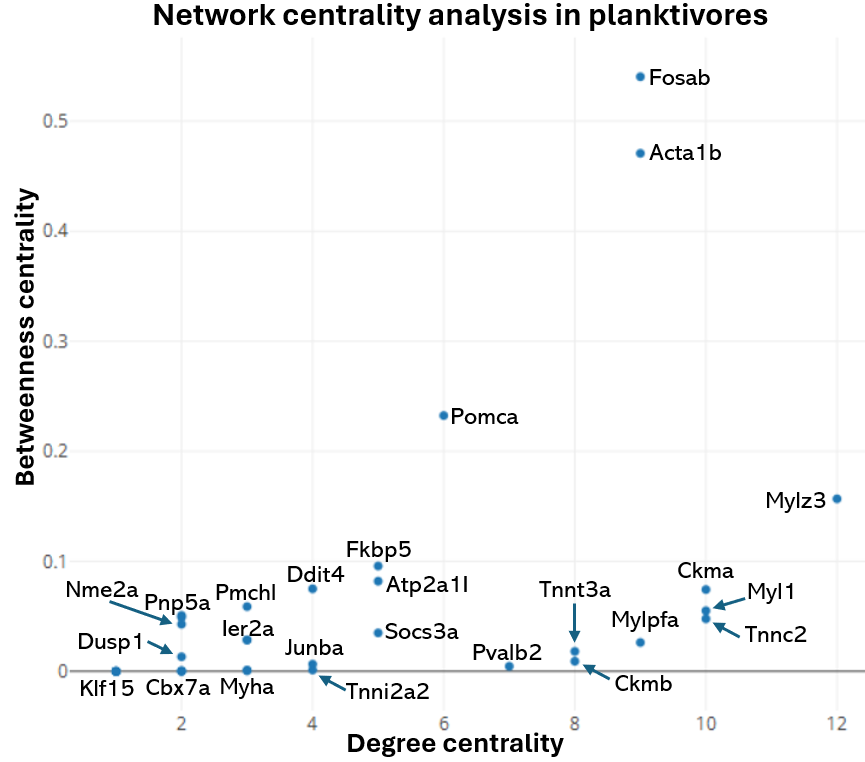
Figure S2** Network centrality analysis in planktivores and planktivores-specific hub genes.

# A characterisation of two potent TFs significantly upregulated in planktivores that were not properly mentioned in the main text follows: ***Fosab*** (v-fos FBJ murine osteosarcoma viral oncogene homolog Ab, in zebrafish aka *cb1065, fos, zgc:77885*). In pikeperch, 2,437 nt long with 4 exons and localized on chromosome 19, *Fosab* → XM_031322272.2 → XP_031178132.1. GO: Function - enables DNA-binding TF activity, RNA polymerase II-specific, enables RNA polymerase II cis-regulatory region sequence-specific DNA binding, Process – involved in regulation of transcription by RNA polymerase II, Component - located in nucleus. Conserved domains: cd14721 (location:126 → 187) bZIP_Fos; Basic leucine zipper (bZIP) domain of the oncogene Fos: a DNA-binding and dimerization domain. Fosab is a part of a bigger Fos family of TFs which includes c-Fos, FosB, Fra-1 and Fra-2 (Milde-Langosch, 2005). The human *c-fos* forms a heterodimer with *c-jun* (part of the Jun family of TFs, also enriched and highly connected in planktivores). The heterodimerization results in the formation of AP-1 (Activator Protein-1) complex binding DNA at AP-1 specific sites, TPA-responsive elements (TRE’s; TGAC/GTCA, Milde-Langosch, 2005) at the promoter and enhancer regions of target genes and converts extracellular signals into changes of gene expression (Chiu et al., 1988). It plays an important role in many cellular functions and has been found to be overexpressed in a variety of cancers. Recently investigated in zebrafish as causing craniofacial anomalies (Maili et al., 2023).

***Junba*** - TF jun-B (Milde-Langosch, 2005), aka AP-1, JunB proto-oncogene, AP-1 TF subunit, JunB proteins are important regulators of cytokine expression and the immune response in skin (Singh et al., 2018). JunB expression in brain is known in mammals <https://www.proteinatlas.org/ENSG00000171223-JUNB/brain> (cf. 15 nearest neighbours based on brain RNA expression: trangelin-2 enriched in piscivores). JunB is a repressor of MMP-9 (matrix metalloprotein-9) transcription in depolarized rat brain neurons (Rylski et al., 2009; MMP-2 enriched and highly connected in piscivores). JunB transcription is known in fish (Fu & Wang, 2020; Kiesow et al., 2015; Ishida et al., 2010). Junb and junb-like (junbl) transcripts induced in response to tissue injury in zebrafish in Ishida et al., 2010. There is no record of jun-B transcription in fish brain.

In piscivores, 29 genes were connected in the gene set enrichment analysis and 20 of them formed the main single network (Figure S3). These genes were subjected to the network centrality analysis assessing betweenness centrality and degree centrality. Fourteen genes that yielded the highest values of these parameters are plotted in Figure S3. These genes involve the six collagen genes and eight genes with products tightly related to collagens and extracellular matrix (ECM). These results further underline the importance of collagens and components of ECM in the pikeperch developing brain and in their piscivory:

**Prolyl 3-hydroxylase 1 (P3h1)** is required for proper collagen biosynthesis, folding, and assembly, localized in endoplasmatic reticulum (Vranka et al., 2004).

**Serpinh1b** (serine protease inhibitor) a collagen-specific chaperone, modulator of collagen structural integrity, ER (Ignatz et al., 2024).

**Lumican (Lum)** a member of the Small Leucine-Rich Proteoglycan (SLRP) family, binds collagen molecules to modulate collagen fibril diameter, localized in extracellular space (Schaefer & Iozzo, 2008; Yeh et al., 2010).

**Matrix metalloproteinase-2 (Mmp2)** a collagenase (aka gelatinase A) responsible for the physiological breakdown of ECM during individual development through collagen degradation (Devarajan et al., 1992).

**Actin alpha 2**, involved in migration of neural stem cells (Zhang et al., 2020, 2022).

**Opticin** belongs to class III of the SLRP family, may noncovalently bind collagen fibrils and regulates fibril morphology, spacing, and organization, localized in ECM (Reardon et al., 2000).

**Fibulin 1 (Fbln1)** interacts with collagens in the ECM, fibulins generally evolved as specialized ECM components for tissues involved in continuous stretching both during embryogenesis and at later stages (Mahajan et al., 2021; Zhang et al., 1997; Timpl et al., 2003)

**Fibronectin 1b (Fn1b)** is an important microfibrillar protein of ECM interacting with fibulin 1 (Timpl et al., 2003).

**Figure S3** Network centrality analysis in piscivores and piscivores-specific hub genes.

**
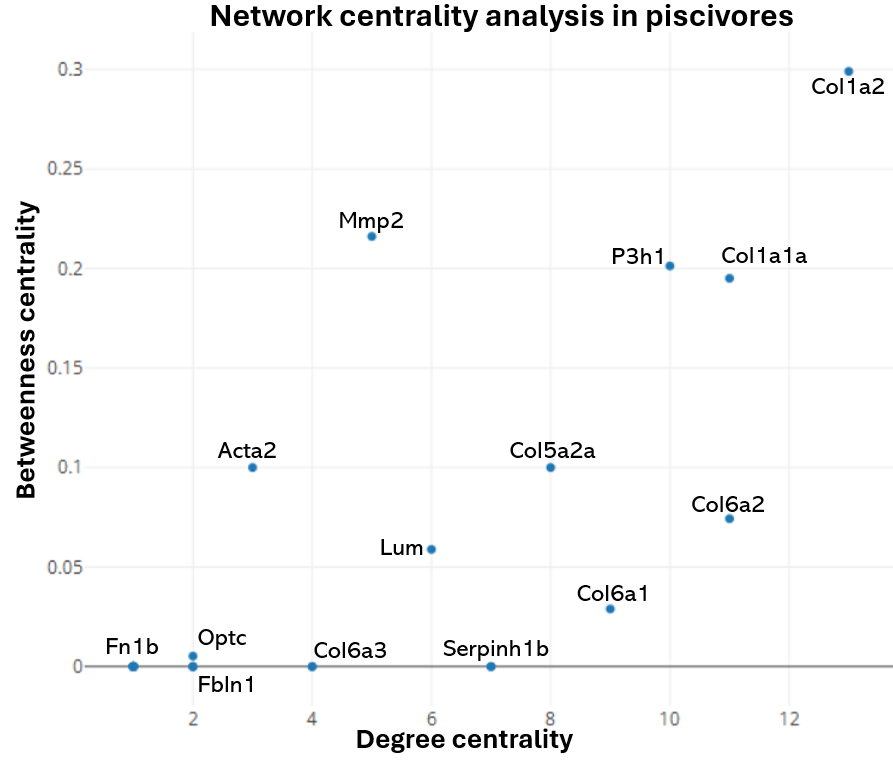
**

**3. Details on snoRNA U85 and its target gene *Ncapd2***

The first snoRNA U85 LOC116049801 resides in intron 5 at the position 1,364 of the gene *Ncapd2*, the second snoRNA U85 LOC116049802 resides in intron 9 (position 4,179), and the third snoRNA U85 LOC116049806 resides in intron 12 (position 5,288; Figure 10 in the main text). The *Ncapd2* gene codes for non-SMC Condensin I complex subunit D2 (LOC116049696, NC_050182.1; SMC = Structural Maintenance of Chromosomes). *Ncapd2* in pikeperch is 41,060,379 nt long and can produce two transcripts of 4,562 nt with 32 exons and 1,399 amino acids (XM_031299602.2, XM_036006195.1). The two *Ncpad2* transcripts differ in three nt in two sites behind the position 150 nt (i.e. >99 % identity).

To test for the potential of snoRNAs U85 to influence the host gene splicing, RNA-RNA interactions were predicted *in silico* between the three snoRNAs U85 sequences and their potentially target gene *Ncpad2* using the IntaRNA 2.0 prediction tool (Mann et al., 2017). The snoRNAs U85 show a significant potential for RNA-RNA interactions with exons of the *Ncapd2* gene (Figure S4): two snoRNAs had predicted interactions with its host gene at the border of two exons, two snoRNAs may potentially interact with a single exon; the first two snoRNAs can potentially interact with exons adjacent to the intron, where they reside (Table 6, Figure 10). Predicted secondary structures of three snoRNA U85 are in Figure S5.

**Figure S4** Minimal free energy (MFE) per query-target pair in snoRNA and Ncapd2. The most significant RNA-RNA interactions between two snoRNAs U85 query sequences LOC116049801 (upper) and LOC116049802 (lower) and the target *Ncapd2* gene as predicted by IntaRNA (Mann et al., 2017).


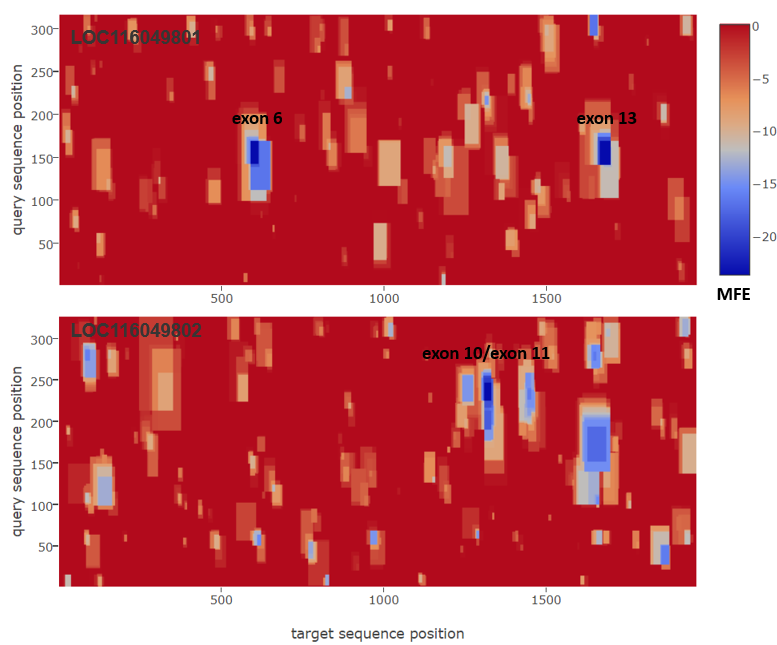


**Figure S5** Predicted MFE secondary structure of three snoRNA U85 upregulated in planktivores. Prediction performed with RNAfold WebServer (Lorenz et al., 2011).


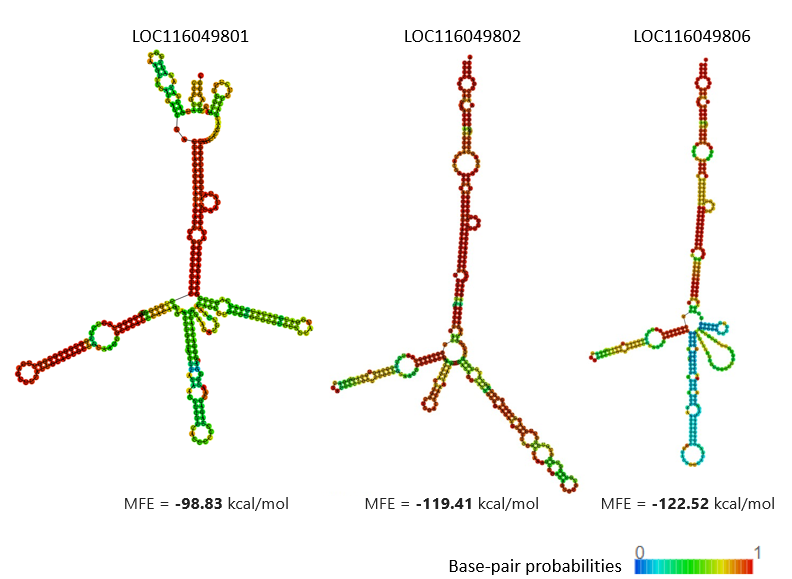


The three snoRNA U85 transcripts were not transcribed equally. Transcripts LOC116049802 and LOC116049806 significantly overrepresented the transcript LOC116049801 (Table 7). The *Ncapd2* gene was, however, transcribed in both pikeperch subcohorts roughly equally. Normalized counts were 306, 241, and 202 for three piscivores and 261, 205, and 234 for three planktivores. The *Ncapd2* gene does not occur in the dataset of differentially spliced genes or differentially transcribed genes.

**snoRNA-mediated regulation of silencing transposon activity?**

The snoRNA U85 is an unusual composite type containing both the C/D and H/ACA boxes and functioning in both 2´-O-ribose methylation and pseudouridylation of snRNA U5 (Jády & Kiss, 2001). The snoRNAs U85 are associated with fibrillarin and Gar1p/dyskerin, which are targets of their above-mentioned domains. Human snoRNA U85 accumulate in subnuclear structures, Cajal bodies, which is in contrast with other snoRNAs accumulating in nucleolus (Darzacq et al., 2002). However, intronic snoRNAs were recently shown to regulate host gene splicing through base pairing with their adjacent sequences (Bergeron et al., 2023), a situation potentially occurring in planktivorous pikeperch analysed here. Since snoRNAs are usually transcribed by RNA Polymerase II, i.e. by the same enzyme synthesizing mRNAs (Kufel & Grzechnik, 2018), it could explain, why the snoRNA U85 transcripts were identified in this study utilizing the poly(A) enrichment. Moreover, some ncRNAs are known to contain poly(A) tails arising through two different ways of polyadenylation (Grzechnik & Kufel, 2008). Regarding the transcript counts and stability of their secondary structure, at least two of here revealed snoRNAs (LOC116049802 and 806) can be considered as plausible results.

The *Ncapd2* gene hosting the three snoRNA U85 in introns, encodes a single subunit D2 of condensin I that is a large mitotic chromosome condensation complex (Watrin & Legagneux, 2005)). Hence, such an isolated single subunit of a large complex can hardly function in the above-mentioned context. In zebrafish, specifically the non-SMC condensin I complex was explored, nonetheless, the Ncapd2 was not detected in a study focused on retina (Seipold et al., 2009). However, a recent study identified specifically Ncapd2 to participate in repression of transposon LINE-1 (Long Noncoding Nuclear Element-1; Ward et al., 2022). This would suggest a fully different function of the snoRNA U85 and its harbouring *Ncapd5* similarly as reported earlier. Namely, snoRNAs can become source of piRNAs, small ncRNA associating with AGO-related PIWI proteins that participate on degradation of transposon RNA (Zhong et al., 2015; Wajahat et al., 2021).

**4. Pathway enrichment analysis with STRING**

Visualization of results on pathway enrichment analysis with STRING version 1.12 <https://string-db.org/> are available below.

**Figure S6** Interaction network of transcripts significantly upregulated in the piscivorous pikeperch subcohort using STRING with the zebrafish (*Danio rerio*) gene annotation (Szklarczyk et al., 2023). The transcripts not included in the network are not shown.


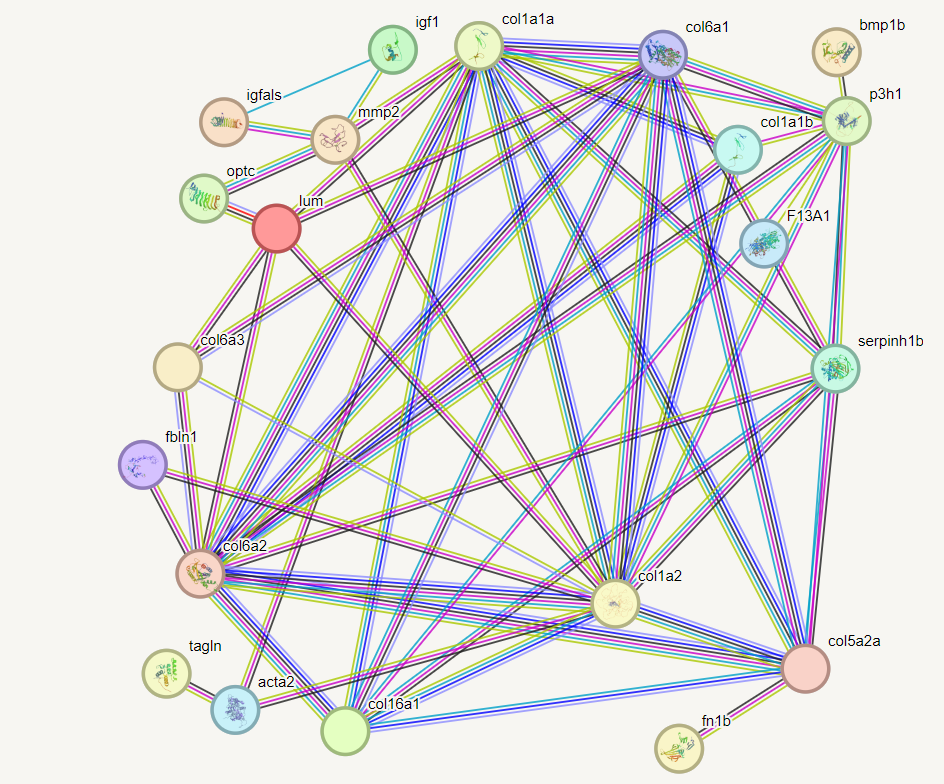

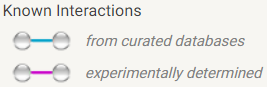

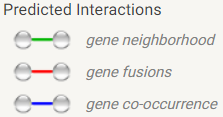

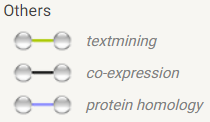


**
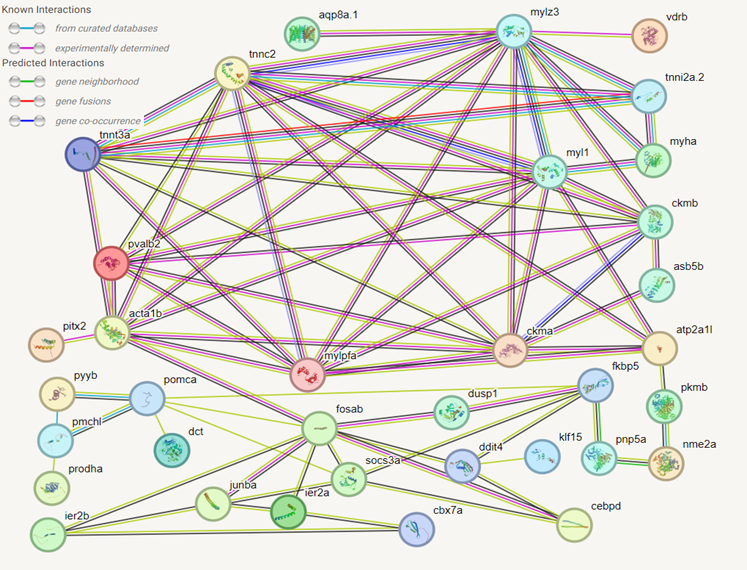
Figure S7** Interaction network of transcripts significantly upregulated in the planktivorous pikeperch subcohort using STRING with the zebrafish (*Danio rerio*) gene annotation (Szklarczyk et al., 2023). The transcripts not included in the network are not shown.

**5. Variants analysis performed on transcriptomics data with CLC Genomics Workbench**

Single nucleotide variants (SNVs) were identified only in the mitogenome (Table S6). There was no variant present specifically in piscivorous nor planktivorous individuals.

**Table S6** Single nucleotide variants (SNV) related to the reference pikeperch mitogenome.

| **Individual** | **pisci1** | **pisci2** | **pisci3** | **plankti2** | **plankti3** | **plankti4** | **SNV type** |
| --- | --- | --- | --- | --- | --- | --- | --- |
| **SNV position** |  |  |  |  |  |  |  |
| 1228 | Y | Y | Y | Y | Y | Y | A to T |
| 2135 | Y | Y | Y | Y | Y | Y | A to T |
| 6938 | Y | Y | Y | Y | Y | Y | G to A |
| 9278 | Y | Y | Y | Y | Y | Y | C to T |
| 10451 | Y | Y | Y | Y | Y | Y | C to T |
| 11556 | Y | Y | Y | Y | Y | Y | C to A |
| 11589 | Y | Y | Y | Y | Y | Y | C to A |
| 12032 | Y | Y | Y | Y | Y | Y | C to T |

# **Supplementary tables (tables S2-S4 are in the Excel file)**

**Table S1** Statistics of mapping the reads to the reference genome of pikeperch

| **Sample ID** | **Total Reads** | **Total Mapped Reads** | **% Total Mapped Reads** | **Unique Mapped Reads** | **% Unique Mapped Reads** |
| --- | --- | --- | --- | --- | --- |
| Piscivor1Brain | 22,474,046 | 22,138,772 | 98.51 | 18,249,761 | 81.20 |
| Piscivor2Brain | 28,257,099 | 27,811,236 | 98.42 | 23,033,036 | 81.51 |
| Piscivor3Brain | 30,891,143 | 30,485,550 | 98.69 | 23,259,224 | 75.29 |
| Planktivor2Brain | 40,188,872 | 39,635,077 | 98.62 | 28,855,296 | 71.80 |
| Planktivor3Brain | 49,720,977 | 49,059,287 | 98.67 | 37,631,296 | 75.68 |
| Planktivor4Brain | 47,858,545 | 47,149,302 | 98.52 | 38,076,666 | 79.56 |
|  |  |  |  |  |  |

**Table S5** Normalized counts of snoRNA U85 transcripts in all pikeperch individuals analysed.

| **snoRNA U85** | **Piscivor1** | **Piscivor2** | **Piscivor3** | **Planktivor2** | **Planktivor3** | **Planktivor4** |
| --- | --- | --- | --- | --- | --- | --- |
| **LOC116049806** | 98.84 | 161.7 | 200.5 | 303.6 | 437.6 | 389.4 |
| **LOC116049802** | 121.4 | 142.4 | 347.2 | 420 | 638.7 | 513.3 |
| **LOC116049801** | 7.06 | 2.41 | 6.8 | 26.3 | 23.9 | 31.9 |

**Supplementary references**

Chiu, R., Boyle, W. J., Meek, J., Smeal, T., Hunter, T., & Karin, M. (1988). The c-Fos protein interacts with c-Jun/AP-1 to stimulate transcription of AP-1 responsive genes. Cell, 54(4), 541–552. <https://doi.org/10.1016/0092-8674(88)90076-1>

Darzacq, X., Jády, B.E., Verheggen, C., Kiss, A.M., Bertrand, E., Kiss, T. (2002). Cajal body-specific small nuclear RNAs: a novel class of 2'-O-methylation and pseudouridylation guide RNAs. *EMBO Journal*, 21(11):2746-56. doi: 10.1093/emboj/21.11.2746

Devarajan, P., Johnston, J. J., Ginsberg, S. S., Van Wart, H. E., & Berliner, N. (1992). Structure and expression of neutrophil gelatinase cDNA. Identity with type IV collagenase from HT1080 cells. *The Journal of biological chemistry*, *267*(35), 25228–25232.

Fu F. & L. Wang, 2020. Molecular cloning, characterization of JunB in *Schizothorax prenanti* and its roles in responding to *Aeromonas hydrophila* infection. Int J Biol Macromol 164

Grzechnik, P., & Kufel, J. (2008). Polyadenylation linked to transcription termination directs the processing of snoRNA precursors in yeast. Molecular cell, 32(2), 247–258. Doi: 10.1016/j.molcel.2008.10.003

Ignatz, E. H., Hall, J. R., Eslamloo, K., Kurt Gamperl, A., & Rise, M. L. (2024). Characterization and transcript expression analyses of four Atlantic salmon (Salmo salar) serpinh1 paralogues provide evidence of evolutionary divergence. *Gene*, *894*, 147984. <https://doi.org/10.1016/j.gene.2023.147984>

Ishida et al., 2010. Phosphorylation of Junb family proteins by the Jun N-terminal kinase supports tissue regeneration in zebrafish. Developmental Biology, 340, 2, 468-479

Jády, B. E., & Kiss, T. (2001). A small nucleolar guide RNA functions both in 2'-O-ribose methylation and pseudouridylation of the U5 spliceosomal RNA. *The EMBO journal*, 20(3), 541–551 [10.1093/emboj/20.3.541](https://doi.org/10.1093/emboj/20.3.541)

Kiesow et al., 2015. Junb controls lymphatic vascular development in zebrafish via miR-182. Scientific Reports. 5, 15007

Kufel J. & Grzechnik, P. (2018). Small Nucleolar RNAs Tell a Different Tale. Trends in Genetics, 35(2): 104-117 doi: 10.1016/j.tig.2018.11.005

Mahajan, D., Kancharla, S., Kolli, P., Sharma, A. K., Singh, S., Kumar, S., Mohanty, A. K., & Jena, M. K. (2021). Role of Fibulins in Embryonic Stage Development and Their Involvement in Various Diseases. Biomolecules, 11(5), 685. <https://doi.org/10.3390/biom11050685>

Maili, L., Tandon, B., Yuan, Q., Menezes, S., Chiu, F., Hashmi, S. S., Letra, A., Eisenhoffer, G. T., & Hecht, J. T. (2023). Disruption of fos causes craniofacial anomalies in developing zebrafish. Frontiers in cell and developmental biology, 11, 1141893. <https://doi.org/10.3389/fcell.2023.1141893>

Milde-Langosch K. (2005). The Fos family of transcription factors and their role in tumourigenesis. European journal of cancer (Oxford, England: 1990), 41(16), 2449–2461. https://doi.org/10.1016/j.ejca.2005.08.008

Reardon, A. J., Le Goff, M., Briggs, M. D., McLeod, D., Sheehan, J. K., Thornton, D. J., & Bishop, P. N. (2000). Identification in vitreous and molecular cloning of opticin, a novel member of the family of leucine-rich repeat proteins of the extracellular matrix. The Journal of biological chemistry, 275(3), 2123–2129. <https://doi.org/10.1074/jbc.275.3.2123>

Rylski, M., Amborska, R., Zybura, K., Michaluk, P., Bielinska, B., Konopacki, F. A., Wilczynski, G. M., & Kaczmarek, L. (2009). JunB is a repressor of MMP-9 transcription in depolarized rat brain neurons. Molecular and cellular neurosciences, 40(1), 98–110. <https://doi.org/10.1016/j.mcn.2008.09.005>

Schaefer, L., & Iozzo, R. V. (2008). Biological functions of the small leucine-rich proteoglycans: from genetics to signal transduction. The Journal of biological chemistry, 283(31), 21305–21309. <https://doi.org/10.1074/jbc.R800020200>

Seipold, S., Priller, F.C., Goldsmith, P. et al. (2009). Non-SMC condensin I complex proteins control chromosome segregation and survival of proliferating cells in the zebrafish neural retina. *BMC Developmental Biology* 9, 40. [10.1186/1471-213X-9-40](https://doi.org/10.1186/1471-213X-9-40)

Singh, K., Camera, E., Krug, L., Basu, A., Pandey, R. K., Munir, S., Wlaschek, M., Kochanek, S., Schorpp-Kistner, M., Picardo, M., Angel, P., Niemann, C., Maity, P., & Scharffetter-Kochanek, K. (2018). JunB defines functional and structural integrity of the epidermo-pilosebaceous unit in the skin. Nature communications, 9(1), 3425. https://doi.org/10.1038/s41467-018-05726-z

Timpl, R., Sasaki, T., Kostka, G., & Chu, M. L. (2003). Fibulins: a versatile family of extracellular matrix proteins. *Nature reviews. Molecular cell biology*, *4*(6), 479–489. <https://doi.org/10.1038/nrm1130>

Vranka, J. A., Sakai, L. Y., & Bächinger, H. P. (2004). Prolyl 3-hydroxylase 1, enzyme characterization and identification of a novel family of enzymes. *The Journal of biological chemistry*, *279*(22), 23615–23621. <https://doi.org/10.1074/jbc.M312807200>

Wajahat, M., Bracken, C. P., & Orang, A. (2021). Emerging Functions for snoRNAs and snoRNA-Derived Fragments. *International journal of molecular sciences,* 22(19), 10193. [doi: 10.3390/ijms221910193](https://doi.org/10.3390/ijms221910193)

Ward, J. R., Khan, A., Torres, S., Crawford, B., Nock, S., et al. (2022). Condensin I and condensin II proteins form a LINE-1 dependent super condensin complex and cooperate to repress LINE-1. *Nucleic acids research*, 50(18), 10680–10694. <https://doi.org/10.1093/nar/gkac802>

Watrin, E., & Legagneux, V. (2005). Contribution of hCAP-D2, a non-SMC subunit of condensin I, to chromosome and chromosomal protein dynamics during mitosis. Molecular and cellular biology, 25(2), 740–750. https://doi.org/10.1128/MCB.25.2.740-750.2005

Yeh, L. K., Liu, C. Y., Kao, W. W., Huang, C. J., Hu, F. R., Chien, C. L., & Wang, I. J. (2010). Knockdown of zebrafish lumican gene (zlum) causes scleral thinning and increased size of scleral coats. *The Journal of biological chemistry*, *285*(36), 28141–28155. <https://doi.org/10.1074/jbc.M109.043679>

Zhang, J., Jiang, X., Zhang, C., Zhong, J., Fang, X., Li, H., Xie, F., Huang, X., Zhang, X., Hu, Q., Ge, H., & Yu, A. (2020). Actin Alpha 2 (ACTA2) Downregulation Inhibits Neural Stem Cell Migration through Rho GTPase Activation. *Stem cells international*, *2020*, 4764012. <https://doi.org/10.1155/2020/4764012>

Zhang, J., Hu, Q., Jiang, X., Wang, S., Zhou, X., Lu, Y., Huang, X., Duan, H., Zhang, T., Ge, H., & Yu, A. (2022). Actin Alpha 2 Downregulation Inhibits Neural Stem Cell Proliferation and Differentiation into Neurons through Canonical Wnt/*β*-Catenin Signaling Pathway. *Oxidative medicine and cellular longevity*, *2022*, 7486726. <https://doi.org/10.1155/2022/7486726>

Zhang, H. Y., Lardelli, M., & Ekblom, P. (1997). Sequence of zebrafish fibulin-1 and its expression in developing heart and other embryonic organs. *Development genes and evolution*, *207*(5), 340–351. <https://doi.org/10.1007/s004270050121>

Zhong, F., Zhou, N., Wu, K., Guo, Y., Tan, W., Zhang, et al. (2015). A SnoRNA-derived piRNA interacts with human interleukin-4 pre-mRNA and induces its decay in nuclear exosomes. Nucleic acids research, 43(21), 10474–10491. [10.1093/nar/gkv954](https://doi.org/10.1093/nar/gkv954)
